# Machine Learning for Structural Steels: Materials Design, Property Prediction, Durability, and Future Directions

**DOI:** 10.3390/ma19173612

**Published:** 2026-08-25

**Authors:** Guomin Wei, Minghe Li, Bo Cui, Wencui Xiu, Asmawan Mohd Sarman

**Affiliations:** 1 School of Mechanical and Civil Engineering, Jilin Agricultural Science and Technology College, Jilin 132101, China; weiguomin@jlnku.edu.cn (G.W.); cuibo888@jlnku.edu.cn (B.C.); xiuwencui@jlnku.edu.cn (W.X.); 2 Faculty of Engineering, University Malaysia Sabah, Jalan UMS, Kota Kinabalu 88400, Sabah, Malaysia; asmawan@ums.edu.my

**Keywords:** structural steels, machine learning, physics-informed machine learning, inverse materials design, service-life prediction

## Abstract

Machine learning (ML) provides new opportunities to model the nonlinear relationships among composition, processing, microstructure, defects, properties, and in-service degradation of structural steels. This structured critical review examines ML applications to materials and process design, microstructural characterization, mechanical-property prediction, corrosion, fire and elevated-temperature performance, fatigue, fracture, and remaining-life assessment. Literature published up to 31 July 2026 was searched primarily through the Web of Science Core Collection and Scopus. A total of 110 publications were retained based on their relevance to structural steels, transparency of data and modeling procedures, and availability of information on validation or engineering applicability. The reviewed studies show that model suitability depends strongly on data modality, sample independence, feature representation, and validation strategy rather than on algorithm family alone. ML has progressed from property prediction toward process optimization, inverse materials design, environmental degradation assessment, and fatigue- and crack-related prognostics. However, independent cross-manufacturer, cross-laboratory, production-scale, and field validation remains limited, while uncertainty quantification and applicability-domain assessment are still inconsistently reported. These limitations are particularly important for corrosion, fire, fatigue, and remaining-life applications, where internally validated models should not be interpreted as substitutes for established physical models or design provisions. Future research should prioritize standardized multimodal data, physics-informed and uncertainty-aware modeling, prospective validation, and rigorously evaluated closed-loop monitoring and digital-twin frameworks for structural-steel life-cycle management.

## 1. Introduction

Structural steel combines high load-carrying efficiency, favorable ductility and toughness, mature fabrication and welding technologies, and substantial recyclability, making it one of the principal load-bearing materials in buildings, bridges, industrial facilities, long-span structures, offshore engineering, and transportation infrastructure. As structural systems move toward greater height, longer spans, increased prefabrication, and extended service life, steel design is no longer governed by strength alone but increasingly requires the simultaneous control of strength, ductility, toughness, weldability, corrosion resistance, fire resistance, fatigue resistance, and cost. These competing requirements have made the design and assessment of structural steels a multi-variable and multi-objective problem. In this review, structural steels primarily refer to conventional carbon structural steels, high-strength low-alloy steels, weathering steels, fire-resistant steels, bridge and pipeline steels, structural stainless steels, and their welded products used in civil infrastructure applications.

The engineering properties of structural steels emerge from coupled relationships among chemical composition, manufacturing history, microstructure, defects, and service conditions. Alloying, steel cleanliness, continuous casting, rolling and cooling schedules, heat treatment, and welding thermal cycles jointly determine phase constitution, grain structure, precipitation, dislocation substructure, texture, inclusions, and local defects, which in turn control strength, ductility, toughness, fatigue resistance, and environmental degradation [1,2,3]. Conventional approaches based on empirical alloy development, physical metallurgy, thermodynamic and kinetic calculations, and numerical simulation provide important mechanistic insight, but become increasingly expensive or restrictive when applied to high-dimensional composition spaces, complex processing routes, multiple performance objectives, and coupled service environments [4,5,6,7,8]. Machine learning (ML) provides a complementary route by learning nonlinear relationships from experimental, computational, industrial, imaging, and monitoring data while retaining the possibility of incorporating physical knowledge.

Applications of ML in steels have consequently expanded well beyond conventional property regression. Current studies encompass phase-transformation prediction, process optimization, microstructure classification and segmentation, defect detection, mechanical-property prediction, multi-objective alloy design, inverse microstructure design, corrosion modeling, elevated-temperature assessment, fatigue and fracture prediction, and remaining-life evaluation [3,9,10,11,12,13,14,15,16,17]. This expansion is accompanied by increasing diversity in data modality, ranging from tabular composition–process datasets and microstructural images to three-dimensional defect data, time-series sensor signals, and physics-based simulations. Accordingly, model performance cannot be judged solely by the choice of algorithm or by a single accuracy metric. The relevance of the input representation, independence of the validation data, physical consistency of predictions, and evidence for transfer beyond the original dataset are equally important for engineering applications.

Existing reviews, however, remain fragmented across these topics. Reviews of ML for alloys and metallic materials have mainly emphasized descriptors, property prediction, microstructure analysis, alloy design, and inverse optimization [4,5,6], whereas specialized reviews have focused on steel microstructures, non-metallic inclusions, constitutive behavior, or fatigue prediction [2,13,14,15,16]. Reviews in construction materials have predominantly addressed concrete and other building materials [17], while AI-oriented reviews of steel structures have concentrated on beams, columns, joints, connections, structural capacity, seismic response, and structural health monitoring rather than on the metallurgical origin of material performance [18]. Reviews closer to steel manufacturing have provided valuable syntheses of process modeling and performance prediction but generally do not connect material design with environmental degradation and service-life assessment [19,20,21,22]. The distinction between these previous perspectives and the present review is summarized in Table 1.

Accordingly, the novelty of this review lies not in cataloguing machine-learning algorithms across steel systems, but in integrating materials design, service degradation, and evidence-strength assessment within a unified materials-to-service framework for civil-infrastructure structural steels.

To maintain a clear scope while still capturing transferable advances from adjacent fields, the evidence considered in this review is classified into three levels. Direct evidence refers to studies on structural steels and welded steel products used in buildings, bridges, pipelines, offshore structures, and related civil infrastructure. Adjacent steel-alloy evidence includes studies on related engineering steels whose metallurgical mechanisms or modeling strategies are relevant to structural-steel applications. Methodological transfer cases include studies on materials such as reduced-activation ferritic–martensitic steels, twinning-induced plasticity steels, nuclear steels, reinforcing steel in cementitious systems, and other non-structural alloy systems and are included only when they demonstrate methods that can reasonably be transferred to structural-steel research. A second distinction is made between prediction scales. The primary scope of the review is the material scale, whereas specimen/joint-, member-, and structural-system studies are included only when they explicitly propagate material degradation, material-state variables, or material-model outputs into engineering performance or life-cycle assessment. This distinction is particularly important in fire, fatigue, and durability applications, where material degradation may propagate across multiple structural scales.

On this basis, the review is organized around three questions: (RQ1) What ML tasks have been investigated across the design, manufacturing, characterization, and service stages of structural steels? (RQ2) What data modalities, feature representations, and validation strategies have been used, and to what extent do they support generalization beyond the original datasets? (RQ3) Which approaches have demonstrated meaningful engineering validation, and what barriers currently prevent the deployment of ML in structural-steel design and life-cycle management? To address these questions, the literature is synthesized according to material relevance, data modality, modeling objective, validation strategy, and engineering evidence, while distinguishing direct structural-steel studies from adjacent evidence and methodological transfer cases. Section 2 describes the review methodology. Section 3 establishes the principal data sources, machine-learning workflow, and trustworthy evaluation criteria. Section 4 reviews composition and process design, microstructure modeling, mechanical-property prediction, and inverse materials design. Section 5 examines corrosion, fire and extreme-temperature performance, fatigue, fracture, and remaining-life assessment. Section 6 synthesizes the current challenges and future research directions, and Section 7 presents the principal conclusions.

## 2. Review Methodology

A structured literature search was conducted to identify studies on machine learning (ML) relevant to the design, manufacturing, characterization, property prediction, durability, and service-life assessment of structural steels. The Web of Science Core Collection and Scopus were used as the primary bibliographic databases, supplemented by targeted searches and backward and forward citation tracking of relevant reviews and primary studies. The literature search covered publications available up to 31 July 2026, with no lower publication-year restriction. Only English-language publications containing sufficient information on the material system, dataset, modeling procedure, or validation strategy were considered. The search combined terms related to computational methods, steel materials, and engineering tasks, including machine learning, deep learning, artificial intelligence, data-driven, structural steel, high-strength steel, weathering steel, pipeline steel, microstructure, property prediction, corrosion, fire, fatigue, fracture, and remaining life.

To avoid interpreting all reported prediction accuracies as equivalent evidence, validation strength was classified into four levels: V1, internal random train–test partitioning or conventional cross-validation; V2, group-based validation using independent heats, specimens, literature sources, or production periods; V3, external validation using an independent laboratory, production line, manufacturer, steel grade, or separately produced heat; and V4, engineering-scale validation involving industrial production, full-scale testing, field monitoring, or operational infrastructure. Because the reviewed studies differ substantially in materials, target variables, dataset structures, and performance metrics, their reported accuracies were not statistically pooled. Instead, the evidence was critically synthesized according to material relevance, data modality, physical representation, validation strength, uncertainty treatment, and experimental or engineering verification. This framework was used consistently in the subsequent comparison of ML applications to structural-steel design, property prediction, durability, and service-life assessment.

## 3. Fundamentals of Structural Steels and Machine Learning

### 3.1. Material Characteristics and Data Sources of Structural Steels

Machine-learning data for structural steels are primarily derived from experimental testing, published literature and open databases, industrial production, materials characterization, in-service monitoring, and numerical simulation. These data sources differ not only in volume and modality but, more importantly, in their degree of statistical independence and physical fidelity. A dataset containing thousands of records does not necessarily contain thousands of independent material states. Repeated specimens from one heat, cropped patches from the same micrograph, dense sensor measurements from one production campaign, and repeated measurements under the same exposure condition may be strongly correlated. Dataset size should therefore be reported together with the number of independent heats, specimens, exposure conditions, or production campaigns rather than using record count alone as an indicator of information content [19,20,21].

Experimental data provide the most direct link to material behavior but are costly to generate for fracture toughness, long-term corrosion, creep, very-high-cycle fatigue, and other time- or resource-intensive properties. Literature-derived datasets can broaden the range of steel grades, processing conditions, and service environments, but introduce heterogeneity associated with grade nomenclature, specimen geometry, sampling orientation, testing temperature, loading rate, test standards, and reporting precision. Industrial datasets may contain very large numbers of process records but considerably fewer independent heats or production campaigns. They are also affected by sensor drift, equipment maintenance, process changes, abnormal operating conditions, and manual-entry errors [19,20,21,22]. Consequently, the effective information content of an industrial dataset depends on both the number of records and the diversity of independent material and process conditions represented.

Steel production generates tabular and time-series variables including chemical composition, temperature, processing time, flow rate, pressure, rolling force, roll gap, and cooling-water flow rate. These data are particularly suitable for endpoint prediction, process optimization, anomaly detection, and quality early warning. However, randomly separating individual records collected from the same heat or production period may result in information leakage. Industrial datasets should therefore be partitioned by heat, production campaign, or chronological production period whenever the intended application involves prediction for future production or previously unseen material batches.

Microstructural and defect data provide an intermediate representation between manufacturing history and macroscopic performance. Optical microscopy (OM), scanning electron microscopy (SEM), electron backscatter diffraction (EBSD), and transmission electron microscopy (TEM) characterize phase constitution, grain structure, crystallographic texture, precipitates, and inclusions, whereas X-ray computed tomography (X-CT) can provide three-dimensional information on internal pores, inclusions, and cracks. For image-based machine learning, the number of cropped patches should be distinguished explicitly from the number of original specimens or independently acquired fields of view. Randomly allocating patches from the same parent image to both training and test sets can lead to severe data leakage and substantially overestimate generalization.

Several open datasets illustrate both the opportunities and limitations of image-based materials learning. The Aachen–Heerlen dataset contains 1705 SEM images and 8909 expert-annotated polygons describing martensite–austenite islands [23]. Its article and associated dataset are persistently identifiable through DOI-based records, facilitating reproducible reuse. The Ultrahigh Carbon Steel Micrograph Database (UHCSDB) links micrographs acquired at different length scales with heat-treatment and microstructural metadata [24]. The ferritic-steel X-CT database provides three-dimensional defect information for mechanically deformed specimens and is accompanied by a persistent Dryad repository record [25]. These resources demonstrate that high-quality labels, specimen-level identifiers, imaging conditions, and access to original rather than only cropped images are critical for reliable reuse.

Data imbalance and censoring require task-specific treatment. Rare brittle-fracture events, severe localized corrosion, extreme inclusions, and very short fatigue lives may constitute a small fraction of a dataset but can dominate structural reliability. They should not be discarded simply because they are statistical outliers. Similarly, fatigue run-outs, particularly in high- and very-high-cycle regimes, are censored observations rather than missing values. Treating run-outs as ordinary failures or removing them from the dataset can bias life predictions. Depending on the task, survival-analysis concepts, censored regression, probabilistic life models, or imbalance-aware sampling should therefore be considered instead of conventional imputation or unrestricted resampling.

Microstructural representation must also retain physically relevant morphology and spatial information. Conventional computer-vision and microstructure-reconstruction studies have demonstrated that phase fraction alone cannot uniquely characterize complex microstructures; morphology, spatial statistics, connectivity, characteristic length scales, and interfacial distributions may contain additional information relevant to mechanical response [26,27]. High-quality labels should therefore reflect metallurgical definitions rather than image contrast alone, and the imaging scale, pixel size, specimen preparation, acquisition conditions, and annotation procedure should be preserved as part of the dataset metadata.

Numerical data are mainly generated through the CALculation of PHAse Diagrams (CALPHAD) method, density functional theory (DFT), molecular dynamics, phase-field modeling, and finite element analysis. Such approaches can efficiently expand the explored composition and processing spaces and provide information on phase stability, microstructural evolution, local stress, and damage variables that may be difficult to measure experimentally. However, computational records should not be pooled indiscriminately with experimental observations. Each numerical dataset should retain an explicit fidelity label and information on governing assumptions, constitutive parameters, boundary conditions, calibration data, and numerical uncertainty.

The discrepancy between simulations and experiments should itself be treated as a source of uncertainty. High- or low-fidelity numerical data can be used for pretraining, surrogate-model development, or multi-fidelity learning, followed by calibration against independent experimental measurements [28]. Uncertainty originating from input parameters, constitutive models, boundary conditions, or numerical approximations should, where possible, be propagated into the final machine-learning prediction. In this context, computational data are most valuable as a source of physical structure and additional coverage rather than as an unquestioned substitute for experimental ground truth. Elemental-composition networks and crystal-graph models likewise demonstrate the potential for automatic representation learning from atomic-scale information, but their application to engineering structural steels still requires scale bridging through processing history, microstructure, defects, and service-state variables [10,11].

As summarized in Table 2, different data modalities provide complementary information. Composition and processing records are efficient for property prediction and process optimization but describe local heterogeneity only indirectly. Microstructural and three-dimensional defect datasets provide stronger links to physical mechanisms but are more expensive to acquire and annotate. Industrial sensors offer high temporal density but may contain relatively few independent production conditions. Simulation can efficiently expand the design space but introduces model-form and parameter uncertainty. Machine learning for structural steels should therefore progress from indiscriminate aggregation of heterogeneous records toward multimodal datasets in which provenance, independence, fidelity, and uncertainty are explicitly represented.

**Figure 1:**
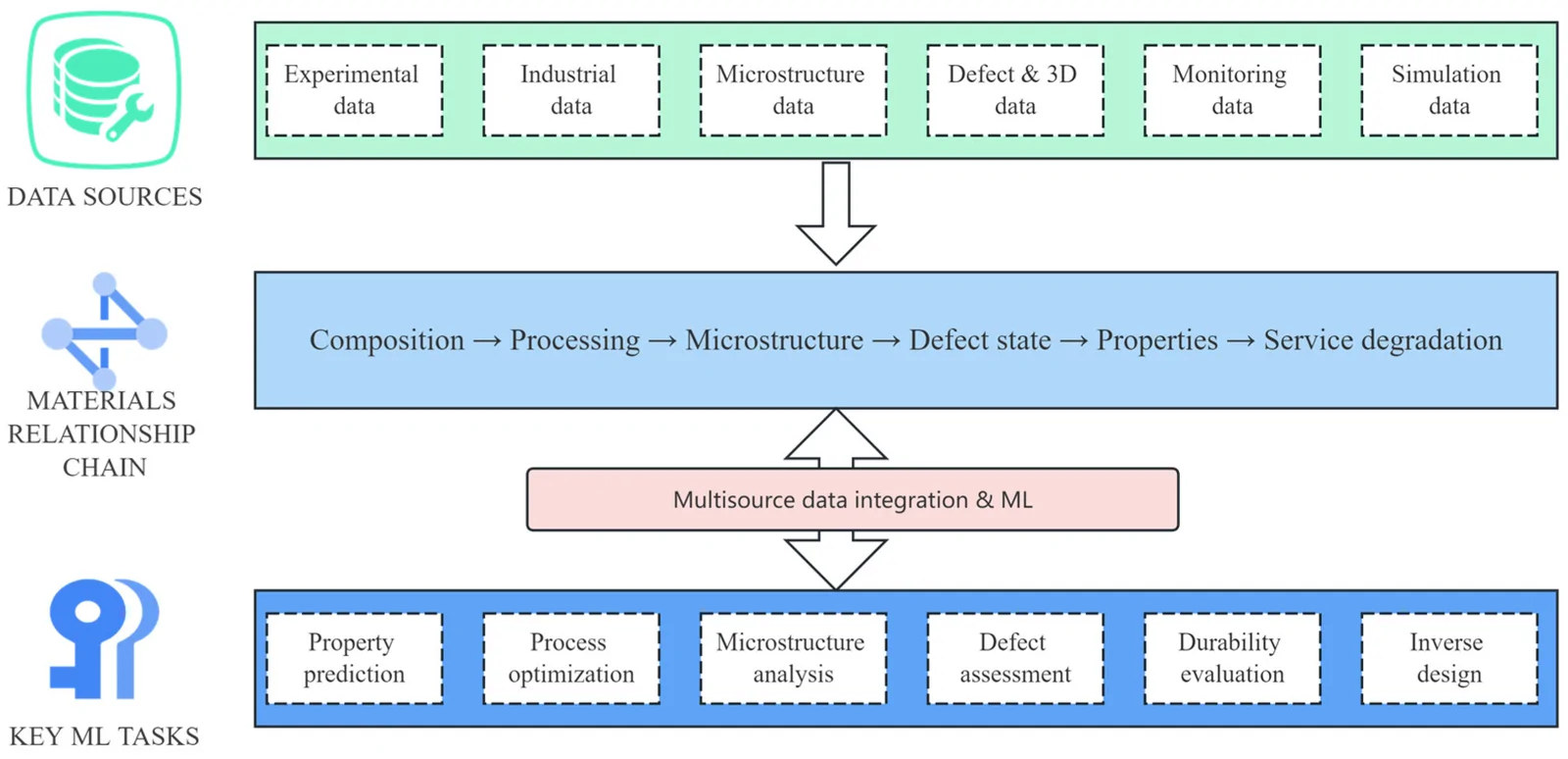
Conceptual framework of multisource data integration and machine-learning applications for structural steels.

Figure 1 follows the materials-science sequence of composition, manufacturing process, microstructure, defect state, macroscopic properties, and in-service degradation. Experimental data, industrial data, microstructural images, sensor signals, and simulation results are linked to property prediction, process optimization, defect identification, durability assessment, and inverse materials design.

Data traceability is essential for reproducibility and model transfer. Structural-steel datasets should, at a minimum, retain the steel grade, heat number, product form, plate or section thickness, sampling location and orientation, manufacturing route, welding procedure, heat-treatment history, specimen geometry, testing standard, loading or strain rate, testing temperature, service environment, characterization equipment, calibration state, preprocessing procedure, and measurement uncertainty. Both data and metadata should conform to the Findable, Accessible, Interoperable, and Reusable (FAIR) principles [29] and maintain a traceable lineage from the original experiment or simulation to the variables used for model training [30]. Without such information, a model may inadvertently interpret batch effects, laboratory practices, equipment differences, or data-processing choices as intrinsic materials relationships.

### 3.2. Machine-Learning Workflow and Model Evaluation

Machine-learning studies of structural steels typically involve problem formulation, data and metadata curation, preprocessing, physics-based feature construction, model training, validation, and engineering deployment. Research in materials informatics has shown that model performance is highly sensitive to descriptor selection, data partitioning, and hyperparameter-optimization procedures. Comparisons among algorithms are therefore meaningful only when they are conducted using consistent datasets and validation protocols [7,8,9,31,32,33,34,35].

To ensure a consistent evaluation framework across different structural-steel applications, this review summarizes machine-learning workflows from data partitioning, model development, performance evaluation, uncertainty analysis, and engineering validation perspectives. These criteria are subsequently used to assess the reliability of reported studies in Section 3 and Section 4.

**Figure 2:**
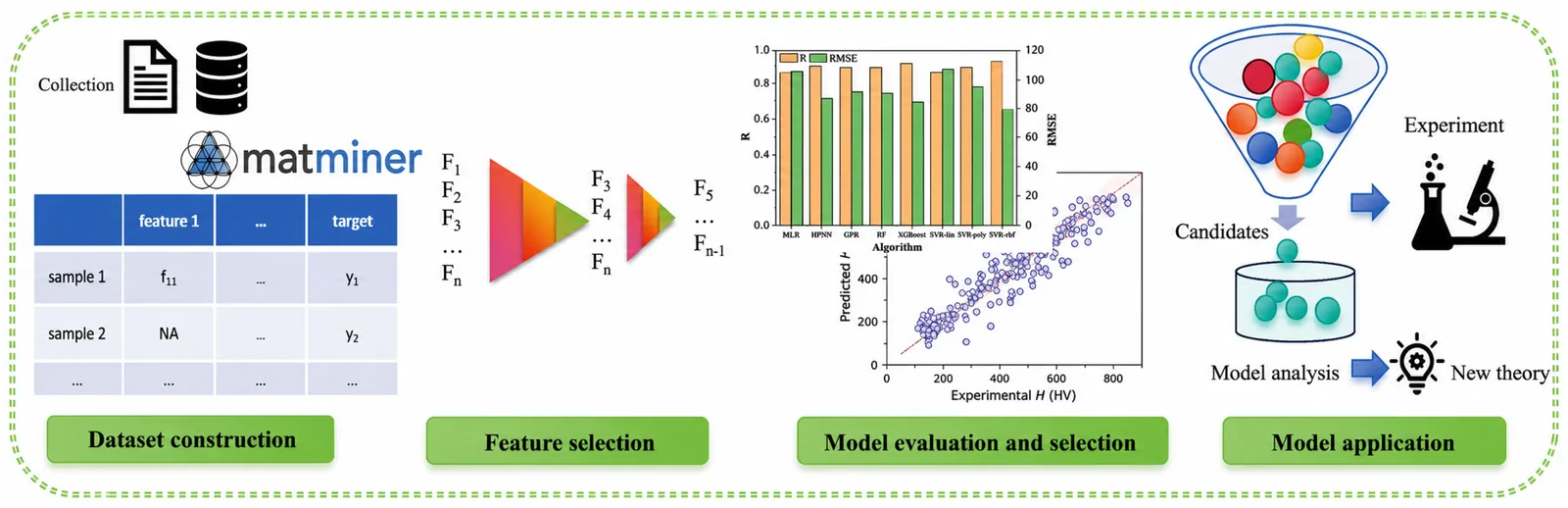
Machine-learning workflow and trustworthy evaluation framework for structural steels [31]. The schematic elements (e.g., feature variables, sample matrix, and candidate points) are used to illustrate general machine-learning procedures rather than specific datasets. Ellipses indicate multiple unshown variables or samples, and different colors are used only for visual distinction.

Figure 2 presents a sequential workflow comprising problem definition, data acquisition and metadata recording, data cleaning, physics-based feature construction, model training and hyperparameter optimization, group-based validation, interpretability analysis, domain-of-applicability assessment, uncertainty quantification, and independent external validation. Particular emphasis should be placed on grouping samples according to heat, steel grade, manufacturer, or production period to prevent data leakage caused by conventional random partitioning.

Data preprocessing requires the standardization of steel-grade designations, composition units, processing variables, and property definitions, together with systematic examination of missing values, duplicate records, and outliers. Anomalous observations should not be removed solely on the basis of statistical thresholds, because low-temperature brittle fracture, exceptionally short fatigue life, or severe corrosion may represent the most engineering-relevant boundary cases. For variables recorded only for specific steel grades or processing conditions, simple mean imputation may also generate artificial material states that do not physically exist.

The purpose of feature construction is not to increase the number of variables, but to establish effective representations of the material state. In addition to raw chemical compositions, physically meaningful variables such as carbon equivalent, hardenability indices, cooling rate, phase fraction, grain size, inclusion size, and defect density may be introduced. General-purpose materials machine-learning frameworks have shown that descriptors derived from elemental properties can increase the information density of composition-based datasets [32]. Open-source tools such as Matminer can further standardize descriptor generation and unify data interfaces [33]. However, large numbers of automatically generated variables may introduce redundancy and multicollinearity. Feature selection must therefore be conducted exclusively within the training data and should not use the complete dataset before the test set has been separated.

The model type should be matched to the sample size and data modality. Random forest, support vector machines, and gradient-boosting models are generally suitable for small- to medium-sized tabular datasets. Convolutional neural networks are appropriate for microstructural and defect images, whereas recurrent neural networks (RNNs) and Transformer architectures are suitable for industrial time-series signals. The principal advantage of deep learning lies in its ability to learn hierarchical representations automatically, rather than simply increasing the number of hidden layers [9,12]. For small-data problems in structural-steel research, physics-based features, regularization, transfer learning, and group-based validation generally improve generalization more effectively than increasing network depth [34].

Data partitioning is among the most frequently overlooked aspects of model evaluation. Different specimens from the same heat, cropped regions from the same image, and repeated tests reported in the same publication are often strongly correlated. If such samples are distributed across both the training and test sets, model performance can be substantially overestimated. Composition–property datasets should therefore be grouped by heat or steel grade, industrial datasets should be divided chronologically, literature-derived datasets should be grouped by source, and microstructural images should be partitioned according to the original specimen. Materials benchmarking studies have demonstrated that differences in data cleaning, partitioning strategies, and hyperparameter optimization can introduce model-selection bias. Model performance is comparable only when consistent datasets and validation procedures are used [35].

In materials informatics, the reported prediction accuracy strongly depends on how training and testing data are separated. Random splitting may lead to overly optimistic estimates when samples from the same heat, production campaign, specimen series, or image source are distributed into both subsets. Therefore, group-based partitioning according to steel grade, heat number, laboratory, production line, or exposure condition is preferred for evaluating engineering generalization. Model performance should be distinguished among three prediction scenarios: interpolation within the known design space, extrapolation within related steel families, and out-of-distribution prediction beyond the original data domain. These scenarios represent different levels of engineering confidence and should not be interpreted using a single test-set metric.

Regression tasks are commonly evaluated using the coefficient of determination (R^2^), mean absolute error (MAE), and root mean square error (RMSE). Classification tasks typically use accuracy, precision, recall, the F1 score, and the area under the receiver operating characteristic curve (AUC). Average metrics alone are insufficient for safety-critical applications. A model may exhibit low overall error within the conventional data range while systematically underestimating brittle-fracture risk, short fatigue lives, or high-temperature instability. Region-specific errors, the proportion of non-conservative predictions, prediction-interval coverage, and calibration error should therefore also be reported.

The selection of evaluation metrics should be consistent with the engineering consequence of prediction errors. For regression tasks involving strength, toughness, corrosion rate, and fatigue life, commonly used indicators include R^2^, RMSE, MAE, and calibration-related measures. However, average errors alone cannot represent safety implications. Underprediction of strength and overprediction of service life may introduce non-conservative risks; therefore, direction-sensitive errors should also be considered. For classification tasks such as failure mode identification or risk assessment, accuracy alone is insufficient, particularly for imbalanced datasets. Balanced accuracy, Matthews correlation coefficient, precision–recall curves, area under the precision–recall curve, and class-specific recall should be reported when appropriate.

High test-set accuracy does not necessarily indicate reliable extrapolation. Domain-of-applicability analysis can identify the composition, microstructure, and processing regions within which a model is expected to maintain relatively low prediction errors [36]. When distribution shifts exist between the training data and a new steel grade or production line, performance obtained from a randomly selected test set may substantially overestimate real-world generalization [37]. Accordingly, leave-one-heat-out, leave-one-grade-out, cross-manufacturer, and cross-laboratory validation provide more meaningful assessments of engineering applicability than merely increasing the size of a random test subset.

When hyperparameter optimization or model selection is performed, nested cross-validation is recommended to avoid information leakage between optimization and evaluation procedures. Otherwise, even group-based validation may retain optimistic bias because model configurations have already been indirectly optimized toward the validation data.

Uncertainty quantification helps determine whether an individual prediction is sufficiently reliable for practical use. Prediction intervals, Gaussian-process models, ensemble methods, and quantile regression can provide risk warnings when an input approaches or exceeds the range represented by the training data [38]. Explainable machine learning can further clarify the contributions of composition, processing, and microstructural variables to model predictions. However, feature-importance measures and SHapley Additive exPlanations (SHAP) values describe statistical associations and do not, by themselves, establish causal mechanisms. Their interpretation must therefore be examined against theories of phase transformation, strengthening, fracture, and corrosion [39]. On this basis, machine-learning models for structural steels should be evaluated comprehensively across the six dimensions summarized in Table 3.

Feature importance methods such as SHAP and permutation importance provide information on model-specific associations rather than direct causal mechanisms. Therefore, interpretation results should be examined for stability across data partitions, model families, and perturbation analyses, and should be further supported by physical understanding or experimental evidence. In addition, uncertainty quantification is essential for safety-critical structural-steel applications. Prediction intervals, calibration analysis, applicability-domain assessment, and out-of-distribution detection should be considered to identify cases where models may produce unreliable predictions.

Overall, the reliability of machine-learning models for structural steels depends first on whether the available data adequately describe the material state, second on the data-partitioning and validation strategies, and only then on the choice of algorithm. High-quality studies should report not only predictive accuracy but also the applicable ranges of steel grades, processing conditions, and service environments, together with physical interpretation, uncertainty estimates, and independent validation results. Only when data are traceable, models are interpretable, and predictions are independently verifiable can machine learning progress from a property-fitting tool to a reliable method for structural-steel design and engineering decision-making.

## 4. Machine Learning Applications in Structural-Steel Design and Property Prediction

The applications discussed in this section are categorized according to the material design and evaluation hierarchy rather than algorithm type. First, machine learning is applied to composition, processing, phase transformation, and microstructure design, where the objective is to establish relationships between manufacturing history and internal structure. Subsequently, models are used for mechanical-property prediction and multi-objective inverse design, where predicted properties are transformed into material-selection strategies. Throughout this section, studies are evaluated not only by predictive accuracy but also by dataset independence, validation strategy, uncertainty treatment, and applicability domain.

The general workflow of machine learning for structural steels involves developing forward models from composition, manufacturing-process, and microstructural data, followed by the integration of physical-metallurgy knowledge and optimization algorithms to infer material compositions, processing schedules, or microstructures that satisfy prescribed performance targets. Compared with studies focused solely on predictive accuracy, data-driven structural-steel design places greater emphasis on the physical plausibility, manufacturability, and experimental verifiability of the predicted solutions.

### 4.1. Composition, Process, Phase Transformation, and Microstructure Design

The studies discussed in this section are classified according to their relevance to civil-infrastructure structural steels. Direct evidence refers to studies using steels explicitly developed or evaluated for structural applications in buildings, bridges, pipelines, or other civil infrastructure. Adjacent-steel evidence includes closely related high-strength, low-alloy, pipeline, or welding steels whose composition–process–microstructure relationships are relevant to structural-steel design. Methodological transfer cases refer to studies on other steel families, such as high-alloy, stainless, or automotive steels, which are included only when their machine-learning strategy provides transferable methodological insight. This distinction is maintained throughout the following discussion to avoid treating methodological similarity as evidence of direct engineering applicability.

Physics-guided modeling provides an important methodological transfer case. Shen et al. [40] incorporated precipitation-related physical descriptors into support-vector and classification models coupled with a genetic algorithm for ultrahigh-strength stainless-steel design. The physical descriptors helped exclude thermodynamically infeasible candidates, although the material system lies outside conventional civil-infrastructure structural steels.

To address the long experimental cycles required to determine time–temperature–transformation (TTT) and continuous-cooling-transformation (CCT) diagrams, Huang et al. [41] combined classification and ensemble regression to predict TTT diagrams for high-alloy steels. The dataset contained 58 TTT diagrams, with five complete steel curves held out for testing, thereby avoiding the direct mixing of the test-steel curves with the modeling data. However, these high-alloy steels are not representative of conventional civil-infrastructure structural steels and are therefore considered a methodological transfer case. Geng et al. developed a hybrid model for synthetic weld heat-affected-zone CCT diagrams of low-alloy steels using 97 complete CCT datasets, with 91 groups for model development and six steels reserved for testing [42]. Because the target was the welding heat-affected zone rather than bulk structural-steel transformation behavior, this study is considered adjacent-steel evidence. Similar hardenability models were developed for non-boron and boron steels using Jominy data [43,44]. These studies demonstrate the value of curve-level prediction for heat-treatment and alloy-design tasks, but their applicability to civil-infrastructure structural steels remains dependent on compositional overlap and validation using independent structural-steel grades.

Recent studies have further integrated transformation-product type, phase fraction, and microstructural morphology within unified modeling frameworks. Cao et al. first transformed reheating and deformation parameters into physically meaningful state variables, such as prior-austenite grain size and stored deformation energy. Gradient-boosted decision trees (GBDTs) and support vector machines were then used to predict the formation and volume fractions of polygonal ferrite, granular bainite, acicular ferrite, and lath bainite. The resulting models were subsequently applied to alloy-lean design and rolling–cooling route optimization for high-strength low-alloy steels. The predicted compositions and processing schedules were subsequently evaluated through newly produced steel and microstructural and mechanical-property characterization [45]. Because the study reports experimental validation of newly designed compositions but does not provide sufficient information to quantify the compositional distance from the training domain or to establish prospective validation in the strict sense, it is treated here as supporting external experimental evidence rather than definitive industrial-scale prospective validation.

This distinction is important because experimental confirmation of a model-generated candidate does not necessarily demonstrate generalization to unseen heats, manufacturers, or production campaigns. In the present review, industrial validation is considered strongest when newly produced heats are independent of model development and their selection is prospectively defined; otherwise, the evidence is classified as external experimental verification but not full production-line validation.

**Figure 3:**
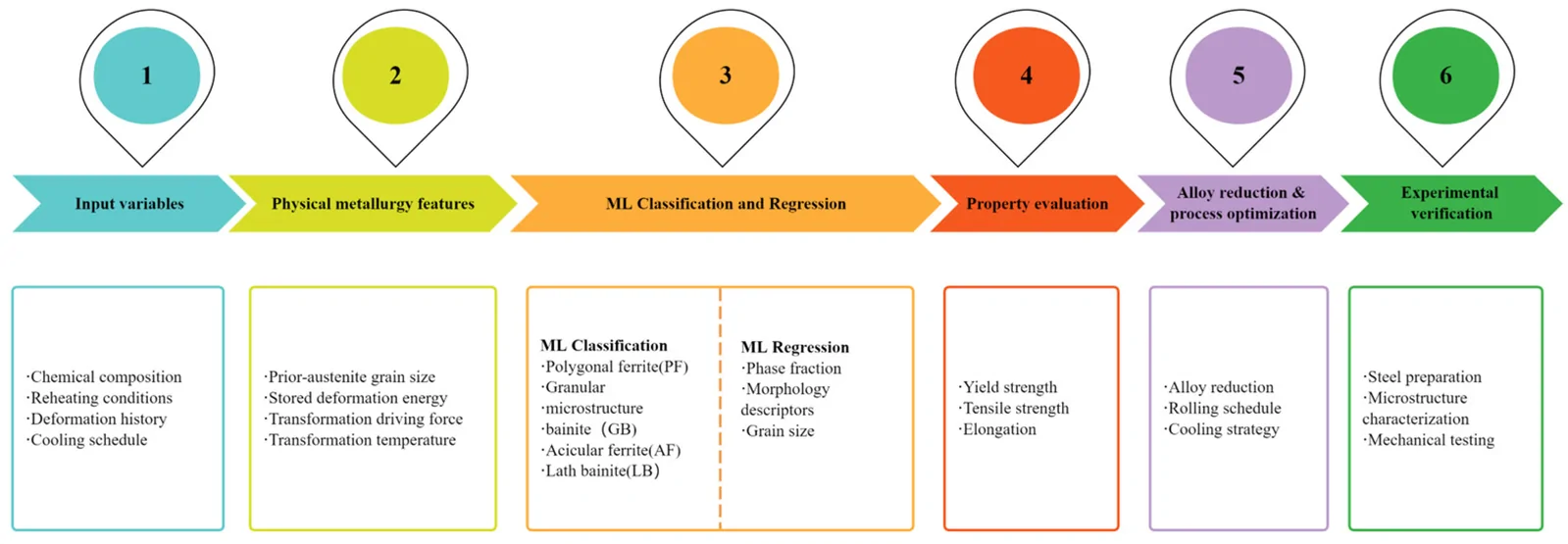
Machine-learning-assisted framework for phase-transformation prediction, alloy-lean design, and process optimization.

As illustrated in Figure 3, this class of approach does not directly predict final properties from processing parameters. Instead, processing variables are first converted into physical descriptors of the transformation state, after which microstructure type, phase fraction, and macroscopic properties are predicted sequentially. The resulting information is then fed back into composition and process design, forming a closed-loop optimization framework.

Microstructural characterization has evolved from manually engineered descriptors toward automatic feature learning. The conventional bag-of-visual-words approach can retrieve and classify microstructures without requiring a predefined feature catalogue, but it remains sensitive to imaging scale, specimen-preparation differences, and local texture variations [26]. Deep neural networks have improved the classification of martensite, tempered martensite, bainite, and pearlite in steels [46]. Nevertheless, image-based performance should be evaluated at the level of original specimens rather than cropped patches, because multiple patches obtained from one specimen are not statistically independent observations. Reliable segmentation also depends on the metallurgical validity and uncertainty of the labels. Studies using electron backscatter diffraction (EBSD) phase maps as supervisory labels can distinguish morphologically similar constituents through crystallographic orientation, grain-boundary characteristics, and intragranular misorientation [47]. However, label uncertainty arising from phase-definition criteria, annotation disagreement, and EBSD indexing quality should be distinguished from model segmentation error. Moreover, high segmentation accuracy does not necessarily imply equally accurate downstream prediction of mechanical properties; these two levels of performance should therefore be evaluated separately. A complementary strategy is to improve segmentation robustness through microscopy-specific pretraining. Stuckner et al. [48] pretrained deep-learning encoders on a large microscopy dataset and transferred the learned representations to microstructure-segmentation tasks, demonstrating improved performance relative to ImageNet-based pretraining, particularly when only limited task-specific training data were available. Although demonstrated using Ni-based superalloys rather than structural steels, this study provides a relevant methodological transfer case for data-limited microstructural analysis.

**Figure 4:**
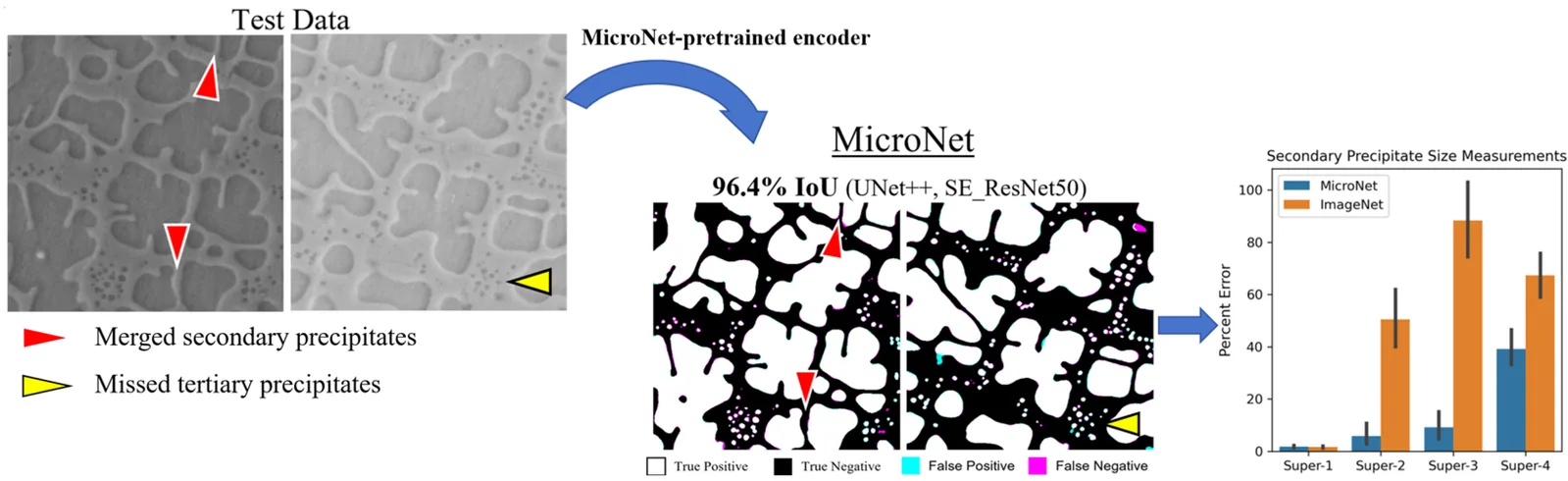
Transfer-learning-assisted deep-learning segmentation and quantitative analysis of complex microstructures [48].

Figure 4 illustrates this microscopy-specific pretraining strategy using representative Ni-based superalloy segmentation results reported by Stuckner et al. [48]. The comparison shows that MicroNet-pretrained models preserve precipitate boundaries more reliably and retain greater sensitivity to fine tertiary precipitates as the amount of task-specific training data decreases, whereas ImageNet-pretrained models exhibit more pronounced over-segmentation and missed detections under few-shot and one-shot conditions. These results highlight the importance of domain-relevant pretraining for microstructure segmentation when experimentally annotated data are scarce. More broadly, however, segmentation accuracy should still be interpreted together with label quality, specimen-level independence, and the accuracy of subsequent quantitative microstructural measurements.

Table 4 summarizes representative applications of machine learning to the composition, processing, and microstructure design of structural steels. The ultimate purpose of microstructure recognition is not merely to produce visually refined segmentation maps, but to establish more accurate microstructure–property relationships. Liu et al. coupled deep-learning-based microstructure segmentation with a random-forest property model and used refined descriptors, including grain size, phase fraction, grain-boundary length, and second-phase interfacial length, to predict the properties of hot-rolled steels. Compared with models based only on average grain size and phase fraction, the RMSE values for yield strength, ultimate tensile strength, and elongation were reduced by 71.53%, 67.51%, and 53.40%, respectively [49]. These results demonstrate that morphological, interfacial, and spatial-distribution information can complement conventional average statistics and improve the representation of strengthening and plastic-deformation mechanisms.

Overall, the available studies can be classified into three technical routes. The first directly predicts phase transformations or microstructures from composition and processing variables. The second introduces intermediate physical variables, such as prior-austenite grain size, stored deformation energy, and thermodynamic quantities. The third automatically extracts microstructural representations from images and transfers them to property-prediction models. The first route is computationally efficient but relies strongly on data coverage when extrapolated. The second offers greater physical consistency but depends on the accuracy of auxiliary models and state variables. The third can capture spatial heterogeneity but remains constrained by labeling quality and imaging conditions. At present, the approach with the greatest engineering potential is not a single algorithm but an integrated framework combining physical state variables, image-based quantification, and industrial validation.

### 4.2. Mechanical Property Prediction

Machine-learning studies of structural-steel mechanical properties should be distinguished according to the target property because tensile strength, impact toughness, and elevated-temperature properties differ substantially in their physical definitions, testing procedures, data scatter, and validation requirements. The following discussion therefore separates room-temperature tensile properties, Charpy impact toughness, and elevated-temperature mechanical properties rather than comparing their prediction accuracy directly.

Room-temperature tensile properties. Yield strength, ultimate tensile strength, and elongation are the most frequently modeled room-temperature properties. Guo et al. analyzed 63,137 cleaned production records from an industrial steel-production database containing 27 composition- and process-related variables and developed simultaneous prediction models for yield strength, ultimate tensile strength, and elongation. The models were subsequently incorporated into a constrained nonlinear-programming framework to identify feasible regions for multiple property requirements [50]. However, the large record count should not be interpreted as an equivalent number of statistically independent material observations. The published study does not report the number of independent heats or production campaigns represented by the 63,137 records, and the validation was based on internal data partitioning rather than independent production-line validation. Accordingly, the reported performance should be interpreted primarily as evidence of interpolation within the represented production domain rather than as proof of cross-plant generalization. Target ranges and measurement uncertainty were also not standardized across the database, which limits direct comparison with independently generated datasets.

For high-dimensional industrial data with relatively few independent material states, introducing physically meaningful intermediate variables can be more effective than simply increasing model complexity. Jiang et al. transformed approximately 100 production parameters for SGLX82A pearlitic steel wire into microstructural descriptors, including proeutectoid ferrite fraction, pearlite fraction, and interlamellar spacing, using thermodynamic, kinetic, and finite-element calculations. The final dataset contained 150 instances after data cleaning. Gradient tree boosting and Gaussian process regression achieved mean relative errors below 0.7% and maximum relative errors below 2.0% under repeated 10-fold cross-validation, and the selected model was additionally evaluated using 10 unseen samples [51]. However, this result should not be interpreted as a universal industrial prediction accuracy because the dataset contained only 150 instances from a single production line and the reported unseen samples did not constitute demonstrated cross-production-line validation. Because the dataset originated from a single production line and a single steel-wire grade, these unseen samples provide evidence of within-domain prediction but should not be regarded as independent cross-production-line validation. The study also demonstrated that multiscale, domain-knowledge-based feature construction reduced prediction errors relative to purely data-based feature-selection strategies; nevertheless, a standardized naive baseline and experimental measurement uncertainty were not reported. The unusually small prediction error should therefore be interpreted in the context of the dataset size, production uniformity, target definition, and validation design rather than as a universal accuracy benchmark.

Physics-guided learning provides another strategy for reducing the dependence on large, labeled datasets. Cui et al. used a strengthening model to generate virtual yield-strength labels for otherwise unlabeled production data, pretrained a deep neural network using these virtual labels, and subsequently fine-tuned the model using experimentally measured data [52]. This approach illustrates the potential of combining mechanistic knowledge with machine learning, but the uncertainty introduced by the virtual labels should be distinguished from experimental measurement uncertainty. The resulting prediction performance is therefore more appropriately viewed as evidence for physics-guided data efficiency than as a direct comparison with purely experimental datasets.

Charpy impact energy represents a distinct prediction task because the measured response depends not only on composition and microstructure but also on specimen geometry, notch configuration, sampling orientation, and test temperature. Wu et al. compared shallow neural networks, extreme learning machines, and deep neural networks for predicting the Charpy V-notch impact energy of low-carbon steel. The Bayesian-optimized DNN achieved a correlation coefficient of 0.9536 and an RMSE of 17.34 J, with the modeled steel having a final thickness of 7.5 mm [53]. These values demonstrate the feasibility of machine-learning prediction within the investigated industrial dataset, but they should not be compared directly with errors from tensile-property models because the target variable, specimen configuration, and test conditions are fundamentally different. In particular, specimen dimensions, orientation, notch geometry, and test temperature should be treated as essential metadata when combining impact-test results from different sources.

Shang et al. further combined industrial production data with literature-derived data for pipeline steel and reported an improvement in the random-forest prediction performance from 0.58 to 0.90 when literature data were incorporated [54]. The result demonstrates the potential of literature-assisted learning to expand a limited industrial dataset, but it also highlights the risk of introducing systematic heterogeneity. Differences in Charpy specimen geometry, notch configuration, sampling orientation, test temperature, and property definitions can produce apparent model improvements that partly reflect changes in data distribution rather than genuine improvement in materials generalization. Therefore, impact-toughness models should be evaluated using harmonized testing metadata and, where possible, source- or specimen-level grouped validation.

Elevated-temperature mechanical properties. Elevated-temperature prediction constitutes a separate task because both the testing path and the definition of the target property influence the measured response. Shaheen et al. compiled 366 elevated-temperature test results from 19 experimental programs covering high-strength structural steels with nominal yield strengths from 460 to 960 MPa [55]. The database included both steady-state and transient-state tests, while heating rate and holding time were recorded when available. The study used chemical composition and temperature to predict the reduction factors for ultimate tensile strength, yield strength, 0.2% proof strength, and Young’s modulus. The reported data showed substantial scatter associated with testing method, manufacturing route, chemical composition, heating rate, holding time, and other incompletely reported experimental conditions. These differences should therefore be considered when interpreting machine-learning performance. In particular, elevated-temperature in-fire properties should not be conflated with post-fire residual properties, because the latter involve a different thermal history and require separate experimental validation.

Peng et al. incorporated CALPHAD-derived transformation temperatures, phase fractions, and precipitate fractions together with experimental composition, heat-treatment, temperature, and prior-austenite-grain-size variables to predict the elevated-temperature yield strength of 9–12Cr steels [56]. This study is included primarily as a methodological transfer case rather than direct evidence for conventional civil-infrastructure structural steels. Its main value lies in demonstrating how thermodynamic and microstructural descriptors can constrain machine-learning predictions and improve physical interpretability. However, the narrow steel-grade range and uncertainties associated with the auxiliary thermodynamic calculations limit direct extrapolation to other structural-steel families.

**Figure 5:**
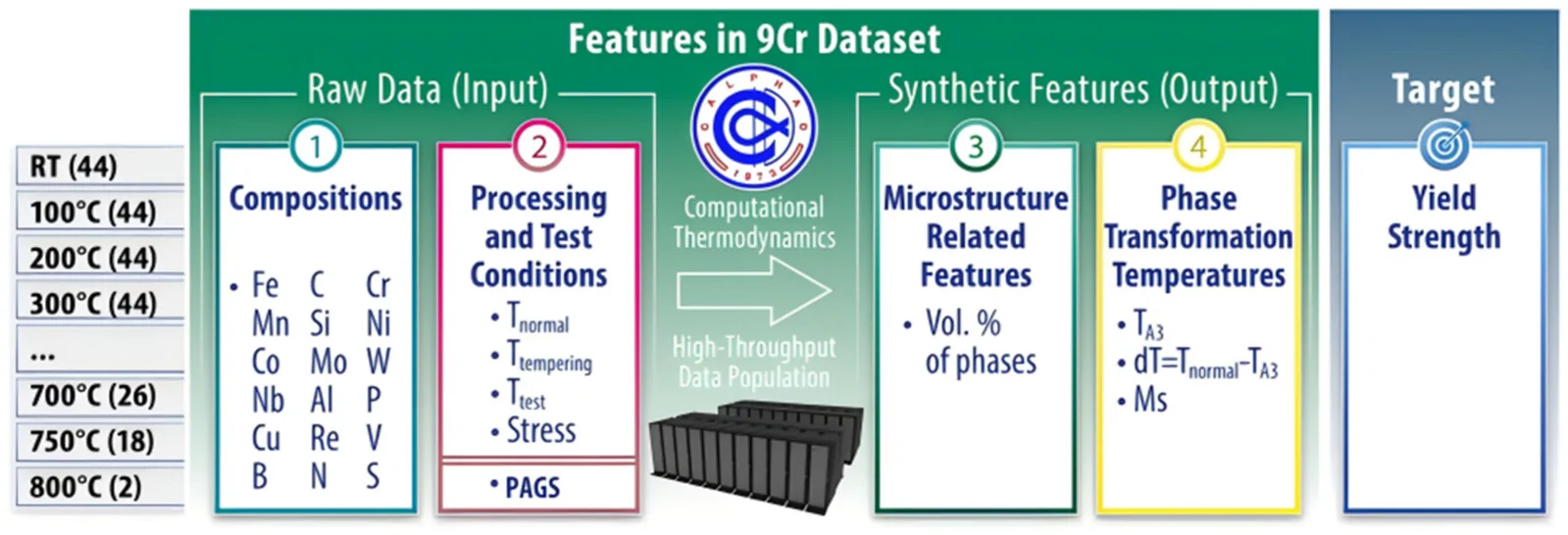
Experimental and synthetic physics-based features used to predict the yield strength of 9–12Cr steels [56].

Across these three property classes, model performance should therefore be interpreted together with the statistical independence of samples, testing conditions, validation type, and uncertainty treatment. High accuracy obtained from randomly partitioned industrial records, repeated measurements from the same production campaign, or heterogeneous literature datasets do not constitute equivalent evidence of engineering generalization. For safety-critical structural-steel applications, external validation across independent heats, production campaigns, laboratories, or testing conditions should be regarded as stronger evidence than a larger internally randomized test set. Representative machine-learning studies for mechanical-property prediction are summarized in Table 5. As a methodological transfer case, Peng et al. incorporated thermodynamic and microstructural descriptors into the machine-learning feature space, as illustrated in Figure 5 [56].

The comparison indicates that reported prediction accuracy should be interpreted together with dataset independence, validation strategy, and testing conditions rather than used as a universal ranking of machine-learning algorithms.

Across these studies, no consistent algorithm ranking can be established because the datasets, target definitions, feature spaces, and validation protocols differ substantially. Tree-based models are frequently competitive for tabular datasets, but their apparent advantages remain dataset- and validation-dependent. The more defensible distinction is therefore between internally validated interpolation and externally demonstrated generalization. However, model rankings are strongly influenced by dataset size, feature construction, and validation strategy. Models trained on large industrial datasets may achieve high internal accuracy while learning production-line-specific correlations. Small-data models can reduce prediction errors by incorporating strengthening theories, CALPHAD-derived features, or pretraining strategies, but they simultaneously introduce uncertainty from the auxiliary physical models or virtual labels. High predictive accuracy therefore cannot replace independent validation across heats, manufacturers, production lines, and testing standards. Results lacking such external evidence should be interpreted as interpolation within a specific dataset rather than as universally applicable materials relationships.

### 4.3. Multi-Property Optimization and Inverse Materials Design

Structural-steel design is usually subject to simultaneous constraints on strength, ductility, toughness, weldability, durability, and cost. Maximizing a single property can therefore cause deterioration in other properties. Diao et al. established models for tensile strength, fracture strength, impact energy, hardness, fatigue strength, and elongation of carbon steels, and used the product of strength and elongation to characterize the strength–ductility balance. Combined with efficient global optimization, this approach illustrates the transition from single-property prediction toward multi-objective materials decision-making [57].

Forward property models combined with intelligent search algorithms have become a major route for composition-based inverse design. Lee et al. developed tensile-strength and total-elongation models using 1075 medium-Mn steel datasets and predicted that Fe–5.5Mn–0.2C–0.3Si steel austenitized at 780 °C could achieve a tensile strength of 1957 MPa and an elongation of 10.7%; the corresponding experimental values were 1952 MPa and 9.9%, respectively [58]. When the database was expanded to 1520 datasets containing microalloying elements and a genetic algorithm was introduced, millions of composition–austenitization combinations could be screened for candidates with tensile strengths above 2GPa. Five experimentally examined steels reached the target strength while retaining useful ductility [59]. These studies demonstrate the feasibility of combining predictive models with optimization, but agreement between predicted and measured properties alone does not establish engineering feasibility.

For engineering-oriented inverse design, candidate materials should additionally satisfy compositional tolerances, feasible processing windows, weldability, cost, and other manufacturing constraints. In particular, an optimized composition located in a sparse region of the training data may have a large prediction uncertainty and may be sensitive to small variations in composition or processing parameters. Therefore, Pareto-optimal candidates should be interpreted as a feasible set rather than a deterministic optimum. The objective functions, constraint boundaries, optimization domain, and treatment of predictive uncertainty should be explicitly considered when ranking candidates. This is especially important for structural steels, for which a small improvement in a target property may not justify reduced robustness or increased manufacturing cost.

Inverse-design targets have also expanded from alloy composition to microstructure. Wang and Adachi established models that predicted stress-strain curves, tensile strength, and total elongation from two- and three-dimensional microstructural descriptors and demonstrated the possibility of inferring microstructural parameters from target properties [60]. However, the inverse mapping from properties to microstructure is generally non-unique: different combinations of phase fraction, morphology, spatial distribution, orientation, and interface characteristics may produce similar macroscopic properties. Consequently, a candidate microstructure generated in a latent space should not be regarded as a directly realizable material unless it can be connected to a feasible composition and processing route.

**Figure 6:**
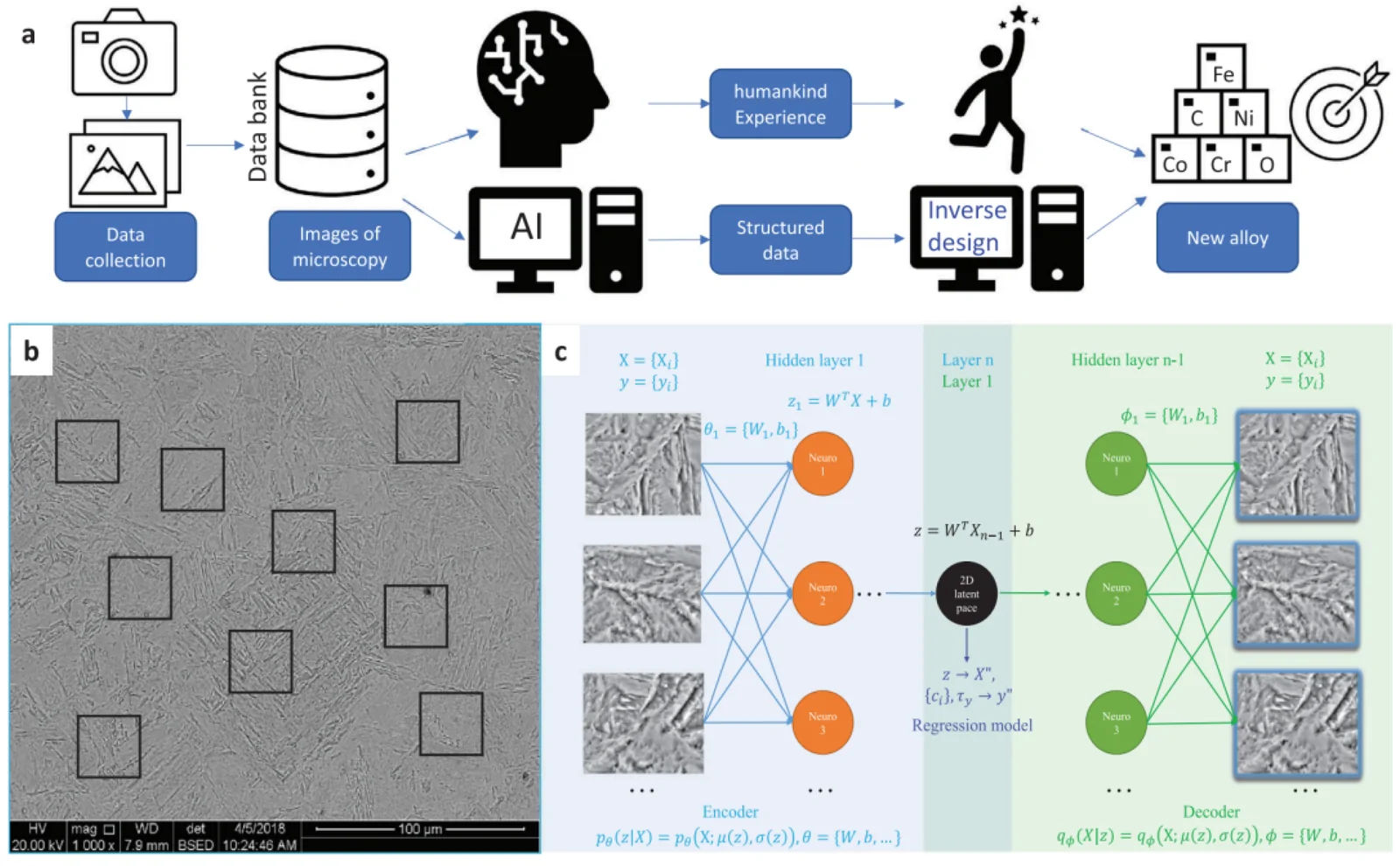
Materials-design approach based on microstructural images and a variational autoencoder [61]. (**a**) General procedure of material design with and without artificial intelligence (AI). (**b**) 32 sub-images of 128 × 128 are randomly chopped from one representative scanning electron microscopy (SEM) image that has a martensitic microstructure. The bottom black bar is excluded from image chopping. (The black squares do not represent the real sizes of sub-images). (**c**) The schematic for the machine-learning model (i.e., VAE) used in this study. The model consists of three sub-models, i.e., the encoder model, the decoder model, and a regression model.

Pei et al. used 621 SEM images of 9–12Cr ferritic-martensitic steels to construct a variational autoencoder-based representation of microstructure. The images were cropped into standardized sub-images, and the encoder, decoder, and regression network were used to extract latent microstructural features, reconstruct images, and predict corresponding alloying-element contents [61]. As illustrated in Figure 6, this approach demonstrates that microstructural images can serve not only as outputs for property interpretation but also as information carriers for inverse alloy design. Nevertheless, the reconstructed microstructure should be distinguished from a manufacturable microstructure, because the latter must also satisfy phase-transformation, processing, and compositional constraints.

Generative models and probabilistic optimization can further improve the exploration of complex microstructural spaces. Kusampudi and Diehl used a variational autoencoder to obtain low-dimensional representations of dual-phase steel microstructures and Bayesian optimization to generate structures with target yield strength and reduced damage sensitivity [62]. Lertkiatpeeti et al. combined representative-volume-element simulations, support-vector regression, neural networks, and Markov-chain Monte Carlo inversion, introducing spatial descriptors such as the Moran index, martensite banding index, and orientation to describe microstructural heterogeneity [63]. Their results demonstrate that identical phase fractions do not necessarily produce identical mechanical properties because phase connectivity, clustering, orientation, and interface distribution also influence the response. These findings further emphasize the non-uniqueness of inverse microstructure–property relationships and the need to incorporate physically realizable processing routes into inverse design.

Inverse-design objectives have also expanded to include cost and service performance. Allen et al. optimized alloy cost while satisfying Jominy hardenability constraints and achieved an average reduction in alloying cost of approximately 18% [64]. Studies of RAFM, austenitic stainless, and TWIP steels have further demonstrated multi-temperature strength–ductility optimization and simultaneous optimization of multiple mechanical properties [65,66]. Wang et al. incorporated strength, ductility, and corrosion resistance of medium-Mn steel within an interpretable optimization framework and experimentally evaluated the resulting candidates [67]. These studies indicate that the objective of multi-property design is not to identify a universally optimal composition, but to construct a set of candidates that simultaneously satisfy performance, physical, manufacturing, and service constraints.

An important distinction should also be made between retrospective and prospective validation. Recovering a previously reported alloy or reproducing a known property represents retrospective validation and mainly tests whether the model can identify solutions already contained in the available knowledge base. By contrast, prospective validation requires the optimization framework to select previously untested candidates, followed by independent material preparation, processing, microstructural characterization, and property testing. The latter provides stronger evidence for the practical value of inverse design because it evaluates the model in a genuinely predictive setting.

**Figure 7:**
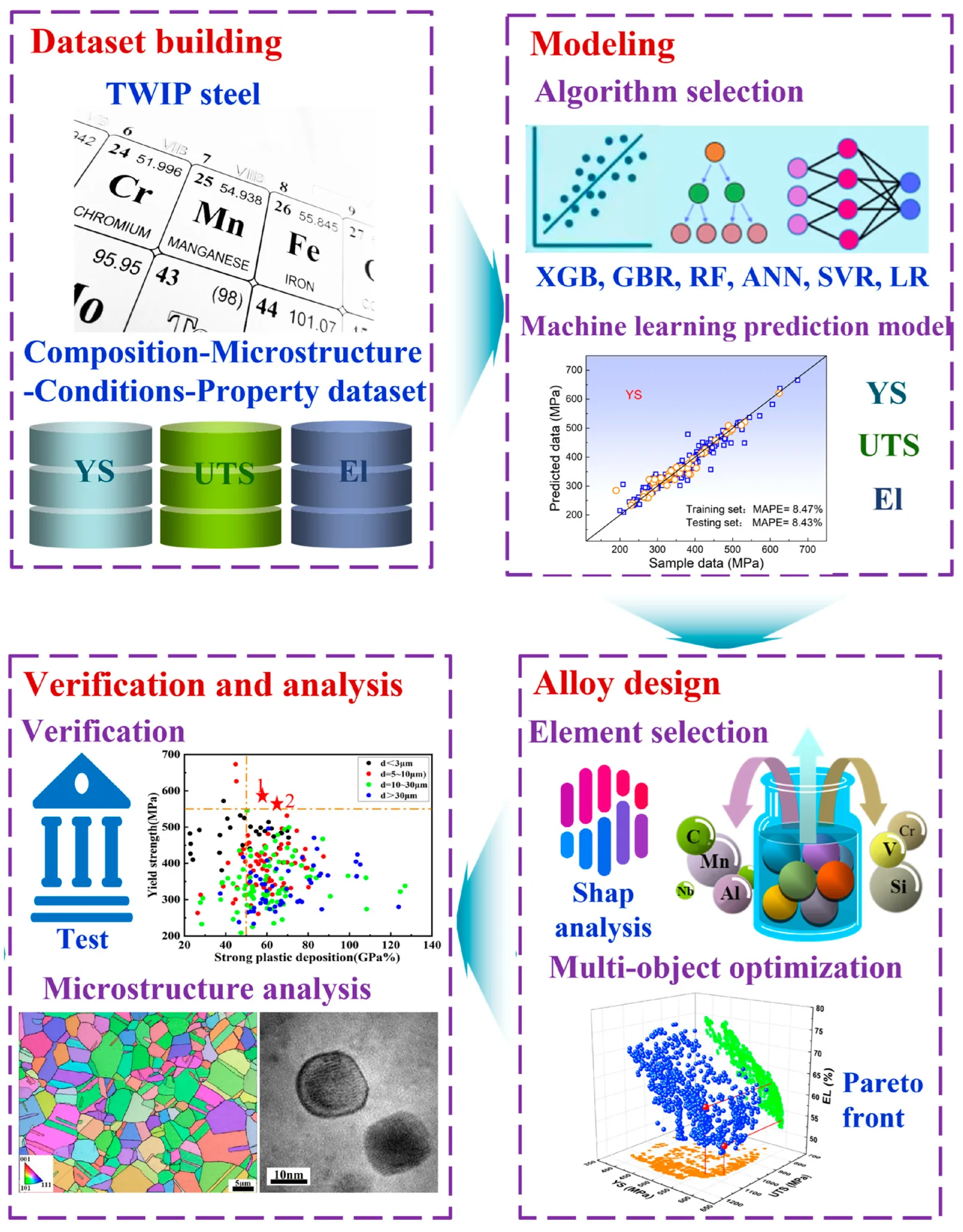
Machine-learning-assisted composition-design workflow for high-yield-strength TWIP steels [68].

Machine-learning-assisted composition design generally consists of data preparation, property modeling, model interpretation, optimization, and experimental verification. Zhou et al. used 273 data entries for TWIP steels containing composition, microstructure, testing-condition, and mechanical-property information to develop prediction models for yield strength, ultimate tensile strength, and elongation. SHAP analysis was subsequently used to identify influential compositional and microstructural variables, followed by multi-objective optimization and experimental validation of newly designed TWIP steels [68]. As illustrated in Figure 7, this framework establishes a closed loop from historical data and property prediction to candidate selection and experimental verification.

Overall, inverse design in structural steels should be viewed as a constrained and uncertainty-aware decision process rather than a direct inversion of a forward prediction model. A reliable workflow should connect target properties with feasible composition, microstructure, and processing routes, quantify prediction uncertainty, and distinguish known-alloy recovery from prospective validation. Such an approach is more consistent with the requirements of structural-steel engineering, where robustness, manufacturability, weldability, cost, and service reliability are as important as the nominally optimized mechanical properties.

## 5. Applications of Machine Learning to Durability and Service-Life Assessment of Structural Steels

Machine learning applications in structural steels have increasingly extended from conventional property prediction to degradation assessment and service-life management. Unlike composition–property modeling, durability-related tasks involve evolving environmental conditions, time-dependent damage, multiple spatial scales, and different levels of engineering evidence. To avoid directly comparing fundamentally different prediction tasks, the studies reviewed in this section are distinguished according to degradation mechanism, target variable, temporal scale, and evidence level.

The principal tasks include corrosion-rate prediction, localized-damage assessment, elevated-temperature property degradation, member-level fire resistance, fatigue-strength and total-life prediction, crack-growth monitoring, and remaining-life estimation (Table 6). At the material level, models mainly predict corrosion rate, retained mechanical properties, or fatigue strength; at the component level, they address member resistance and crack evolution; and at the service level, they estimate damage progression, failure probability, or remaining life. Accordingly, reported prediction accuracy should be interpreted together with the physical target, observation period, validation strategy, and applicability domain rather than used to establish a universal ranking of machine-learning algorithms.

For corrosion and environmental degradation, particular attention is required to distinguish average or instantaneous corrosion from localized damage. Mass loss, corrosion current density, and average penetration rate describe overall degradation, whereas maximum pit depth and pit morphology are more directly related to local stress concentration and structural reliability. These quantities are therefore not interchangeable. Similarly, corrosion under high-temperature chemical environments is considered here as an environmental degradation process, whereas the thermomechanical response of structural steel during fire exposure is addressed separately in Section 5.2.

### 5.1. Corrosion and Environmental Degradation

Corrosion of structural steels is governed by the coupled effects of material composition, surface condition, environmental exposure, and time. Machine learning is particularly useful because it can simultaneously process material, environmental, and temporal variables and capture nonlinear interactions that are difficult to represent using conventional empirical models. However, corrosion prediction studies use substantially different target variables, including instantaneous corrosion current, average corrosion rate, corrosion current density, corrosion depth, and remaining service life. These quantities describe different stages or aspects of degradation and should therefore not be directly compared by their numerical prediction accuracy.

Early studies focused on short-term, continuously monitored atmospheric corrosion. Pei et al. monitored carbon steel using an Fe/Cu galvanic corrosion sensor for 34 days and used relative humidity, temperature, rainfall, airborne particles, and gaseous pollutants to predict instantaneous atmospheric corrosion. Random forest outperformed artificial neural networks and support vector regression, while explicitly considering rust formation further improved prediction. The study demonstrates the value of time-resolved sensor data but represents a relatively short exposure period and primarily describes the sensor-level corrosion response rather than long-term structural material loss [69].

Long-term atmospheric studies have incorporated both alloy composition and exposure-site information. Yan et al. used 306 corrosion records covering 18 alloy steels exposed at three marine-atmospheric sites for 1, 2, 3, 5, 7, and 10 years. The target was corrosion rate expressed as annual corrosion depth (μm·a^−1^). An optimized random forest model achieved R^2^ values of 0.94 and 0.73 for the training and testing sets, respectively [70]. These results demonstrate the importance of jointly considering material, environmental, and exposure-time variables, but the relatively small number of independent alloy instances means that the nominal record count should not be interpreted as equivalent to independent material observations.

The environments experienced by actual structures are rarely stationary. Song et al. investigated dynamic atmospheric corrosion under vehicle operating conditions by combining meteorological and pollution data with operational parameters, including average vehicle speed and the ratio of moving to stationary time. A time-weighting method was used to reconcile variables recorded at different sampling frequencies. Genetic-algorithm-optimized support vector regression achieved an R^2^ value of 0.9771, outperforming both the unoptimized support vector regression model and neural-network models [71]. Liu and Li predicted the corrosion rate of 3C steel in seawater using temperature, pH, dissolved oxygen, salinity, and oxidation–reduction potential as input variables. SHAP analysis identified oxidation–reduction potential as the dominant predictor [72]. These studies indicate that machine-learning-based corrosion analysis has progressed from fitting average environmental conditions toward modeling dynamic service scenarios and providing local explanations of individual predictions.

The application scope of corrosion prediction has also expanded from atmospheric and seawater environments to underground structures and steel–concrete systems. Dong et al. used 1428 experimental records to predict the corrosion current density of steel buried in soil. The inputs included exposure duration, moisture content, pH, electrical resistivity, chloride content, sulfate content, and total organic carbon. Random forest achieved an R^2^ value of 0.987, and soil resistivity, exposure duration, and total organic carbon formed the most effective three-variable combination [73].

**Figure 8:**
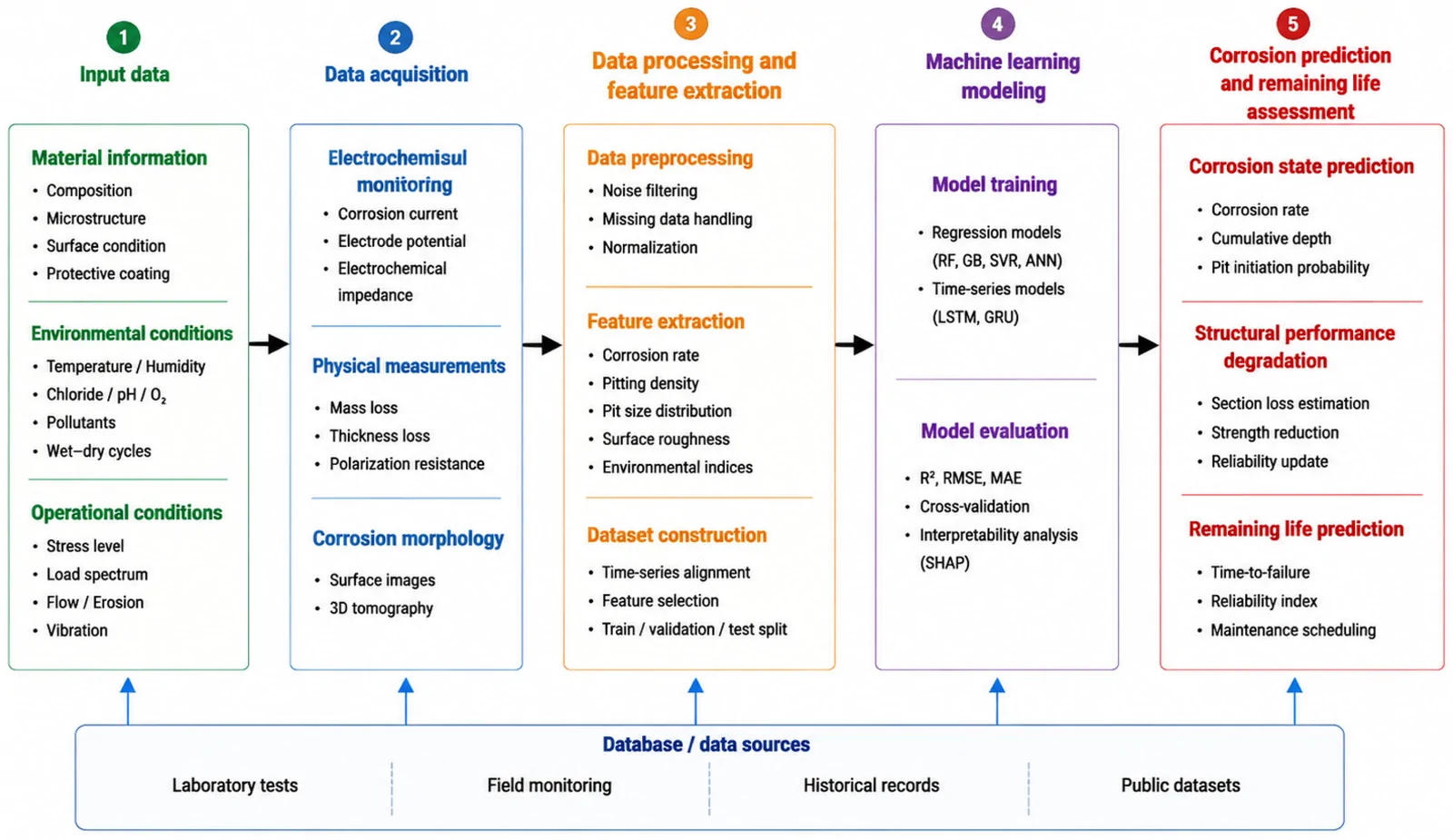
Machine-learning framework for evaluating corrosion degradation and service life of structural steels.

Corrosion studies of reinforcing steel embedded in carbonated cementitious materials do not constitute primary evidence for structural-steel applications, but their treatment of multiphase material parameters, pore-solution chemistry, and environmental humidity provides transferable methodological insights. Ji and Ye identified electrical resistivity, the [Cl^−^]/[OH^−^] ratio, cement proportion, and corrosion potential as critical variables [74]. However, the resulting model cannot be directly extrapolated to exposed structural steel or reinforcing steel in non-carbonated concrete. This limitation demonstrates that corrosion models must explicitly define the substrate, surface condition, and environmental boundaries within which they are valid. Figure 8 summarizes the general workflow from structural-steel corrosion data to service-life assessment.

Figure 8 presents a sequential framework comprising material information, environmental and operational conditions, corrosion monitoring, corrosion-state prediction, and structural service-life assessment. Material information includes chemical composition, microstructure, surface condition, and protective coatings. Environmental variables include temperature, humidity, chloride deposition, pH, pollutants, and wet-dry cycling. Monitoring data may include mass loss, corrosion current, electrical resistance, wall thickness, and corrosion morphology. The model outputs progress from instantaneous corrosion rate and cumulative corrosion depth to cross-sectional loss, load-carrying-capacity degradation, and remaining service life.

Under high-temperature molten-salt, oxidation, and thermochemical environments, corrosion is additionally coupled with temperature, alloy composition, corrosive medium, and exposure duration. Muthukrishnan et al. compiled high-temperature corrosion data for structural materials and protective coatings and compared multiple regression methods. Random forest generally exhibited favorable performance. However, the study also emphasized that currently available data do not adequately cover different molten-salt chemistries, temperature ranges, and material states. The resulting models are therefore more appropriate for preliminary material screening than for replacing long-term corrosion testing [75].

The engineering objective of corrosion prediction is to translate environmental conditions and corrosion states into estimates of cross-sectional loss, structural reliability, and maintenance timing. Ensemble models based on pipeline-accident records and a physics-informed neural network (PINN) developed for marine steel pipe piles represent two different routes for remaining-life prediction [76,77]. The former depends strongly on the representativeness of historical accident databases, whereas the latter relies on assumptions concerning uniform corrosion and boundary conditions. Neither approach yet fully captures the combined effects of extreme pitting, scour, and corrosion fatigue.

**Figure 9:**
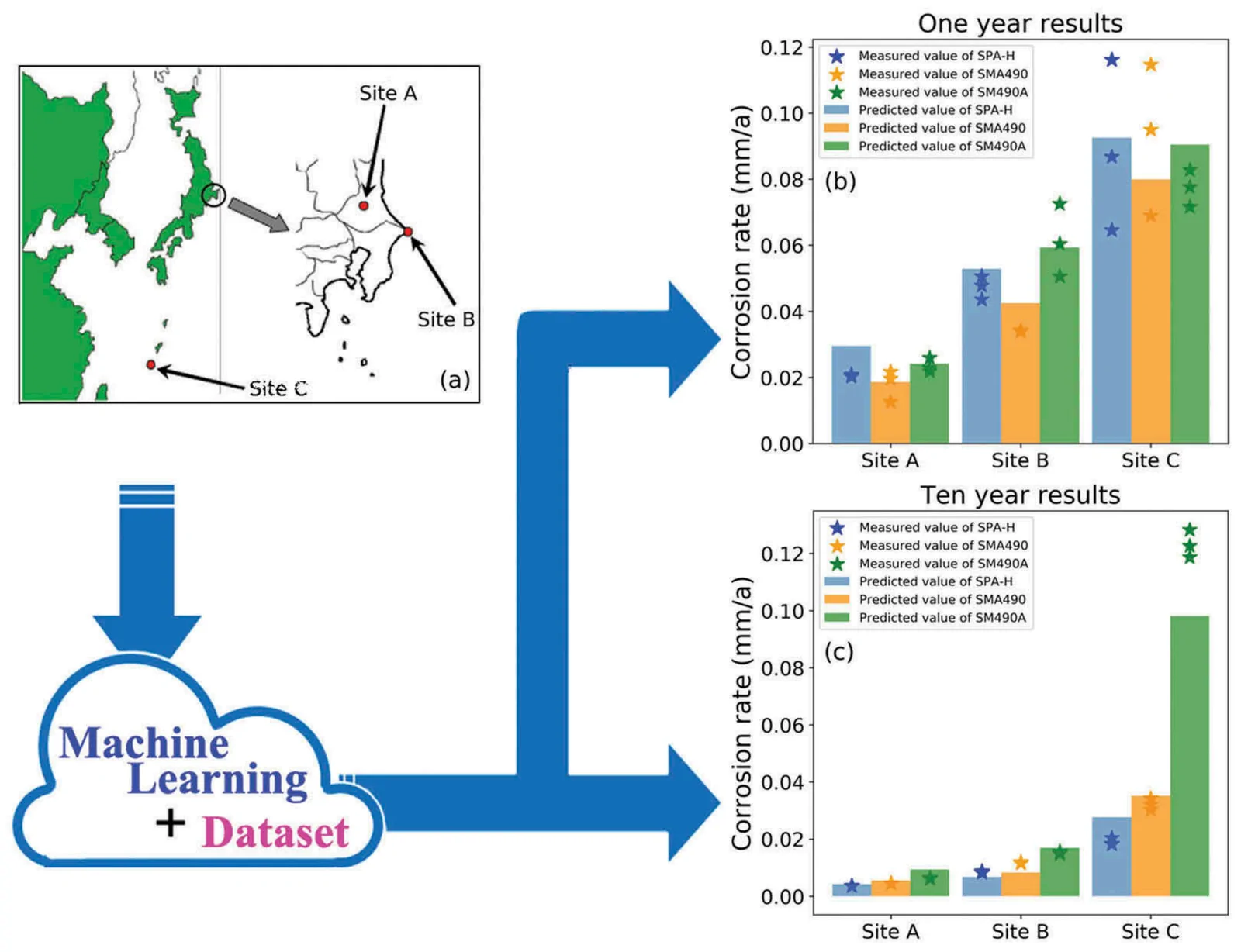
Measured and machine-learning-predicted long-term corrosion rates of low-alloy steels in different marine atmospheric environments [70]. (**a**) illustration of three different atmospheric exposure sites; the measured corrosion rate (star icons, three parallel samples) vs. predicted corrosion rate (bar plot) of typical low-alloy steel SPA-H, SMA490 and SM490A after (**b**) one and (**c**) ten years of exposure.

Feng et al. incorporated chloride diffusion, corrosion depth, wall-thickness loss, and mechanical-property degradation into a PINN for service-life prediction of marine steel pipe piles. After particle swarm optimization, the deviation between the inversely identified diffusion coefficient and the experimental value was 4.3% [77]. Nevertheless, the model approximated the chloride penetration front as a uniform corrosion front. Its predictions therefore primarily represent average wall-thickness degradation and do not fully capture pitting corrosion, crevice corrosion, or corrosion-scour interactions. Figure 9 and Table 7 present, respectively, the measured and machine-learning-predicted long-term corrosion rates of low-alloy steels and additional representative studies on corrosion and environmental degradation of structural steels.

Across exposure classes, the prediction targets and validation strategies differ substantially, making direct comparisons of reported accuracy inappropriate. Short-term atmospheric studies mainly address instantaneous electrochemical responses, whereas long-term atmospheric and soil studies focus on average corrosion rates. Service-life models further transform corrosion degradation into wall-thickness loss or failure time. These tasks should therefore be evaluated according to their physical target, temporal scale, validation design, and engineering consequence rather than by a single accuracy metric.

### 5.2. Fire and Extreme-Temperature Performance

Fire and extreme-temperature performance involves a hierarchical transfer from material degradation to member resistance and finally to structural or infrastructure-level consequences. Temperature-dependent thermal and mechanical properties determine the constitutive response of steel; these properties, together with geometry, loading, thermal gradients, and boundary conditions, determine member stability and fire resistance; member failures can subsequently affect structural robustness and infrastructure-level fire risk. Member- and infrastructure-scale studies are therefore included because they demonstrate how material degradation is translated into engineering consequences. However, they are not treated as direct evidence of material behavior, and their datasets and performance metrics are not directly compared with material-level models.

Predicting temperature-dependent material properties represents only the first level of machine-learning-based fire assessment. Naser used ANN and genetic algorithms to integrate experimental data and material models from fire codes and standards, including ASCE, Eurocode and British Standards, to derive temperature-dependent thermal and mechanical properties of structural steel [78]. The predicted quantities included thermal conductivity, specific heat, yield strength, and elastic modulus or their reduction factors. The study demonstrates that machine learning can reconcile differences among heterogeneous material models, but the resulting expressions remain dependent on the definitions and testing assumptions contained in the source data.

The transferability of temperature-dependent material models is strongly affected by the experimental protocol. Steady-state and transient-state tests do not necessarily produce identical stress–strain responses, while heating rate, strain rate, thermal exposure duration, cooling regime, and post-fire testing conditions can influence the measured properties [78,79]. Accordingly, room-temperature-to-elevated-temperature prediction, transient fire response, and post-fire residual-property prediction should be regarded as distinct tasks rather than a single temperature–property mapping.

The next evidence level concerns structural members, where temperature-dependent material properties interact with geometry, slenderness, loading, eccentricity, boundary conditions, and instability modes. Zhao developed a hybrid neural network optimized by a genetic algorithm to predict the failure temperature of steel columns, incorporating geometric and loading parameters [80]. The study demonstrates that machine learning can capture the combined influence of material and member parameters, but comparison with analytical design equations remains essential because a lower statistical error does not automatically imply a safer engineering prediction.

Possidente and Couto used 21,879 geometrically and materially nonlinear finite-element simulations to train neural-network, random-forest, and support-vector-machine models for compressed L-, T-, and X-shaped steel members at elevated temperatures [81]. The large numerical dataset enables efficient surrogate prediction, but the samples are simulation-derived rather than independent fire-test observations. The models therefore provide a useful numerical proxy while retaining the assumptions and model-form uncertainty of the underlying finite-element simulations. The study itself emphasizes the trade-off between accuracy and safety and shows that applicability is limited by the section shapes and heating conditions represented in the training data.

**Figure 10:**
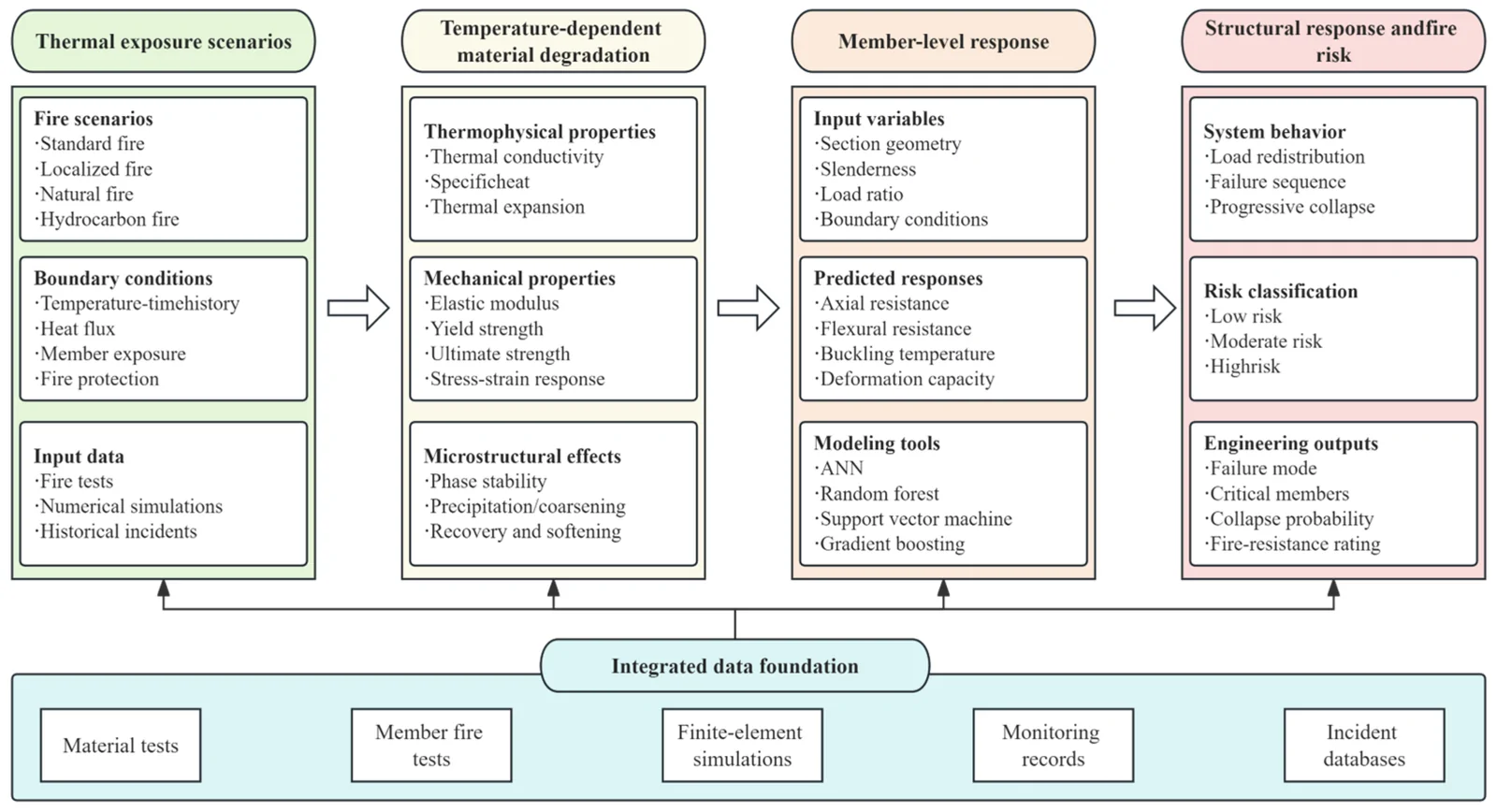
Multiscale machine-learning framework for evaluating the fire and extreme-temperature performance of structural steels.

These member-level models demonstrate that algorithm selection for safety-critical applications cannot be based solely on average error metrics. A model may achieve a low overall root mean square error while still producing non-conservative predictions in regimes involving high temperatures, large slenderness ratios, or torsional buckling. Machine-learning models for member fire resistance should therefore report prediction bias within critical regions, the proportion of unconservative estimates, and quantile-based errors, while also considering the safety margins embedded in design codes. Figure 10 summarizes the multiscale prediction process from material degradation to structural fire risk.

Figure 10 sequentially links fire scenarios and temperature histories, degradation of thermal and mechanical properties, member stability and resistance, structural failure modes, and fire-risk classification. Data at the different levels are derived from elevated-temperature material tests, finite element simulations, member fire tests, and historical fire-incident records.

Machine learning has also been applied to global failure and progressive-collapse assessment of steel structures. Fu used Monte Carlo simulation and random sampling to generate data on fire scenarios, member temperatures, load ratios, and critical temperatures. Decision trees, k-nearest neighbors, and artificial neural networks were then compared for classifying the failure modes of steel-frame members. The predicted member failures were subsequently used to remove failed components from the structural model and assess the likelihood of progressive collapse [82].

**Figure 11:**
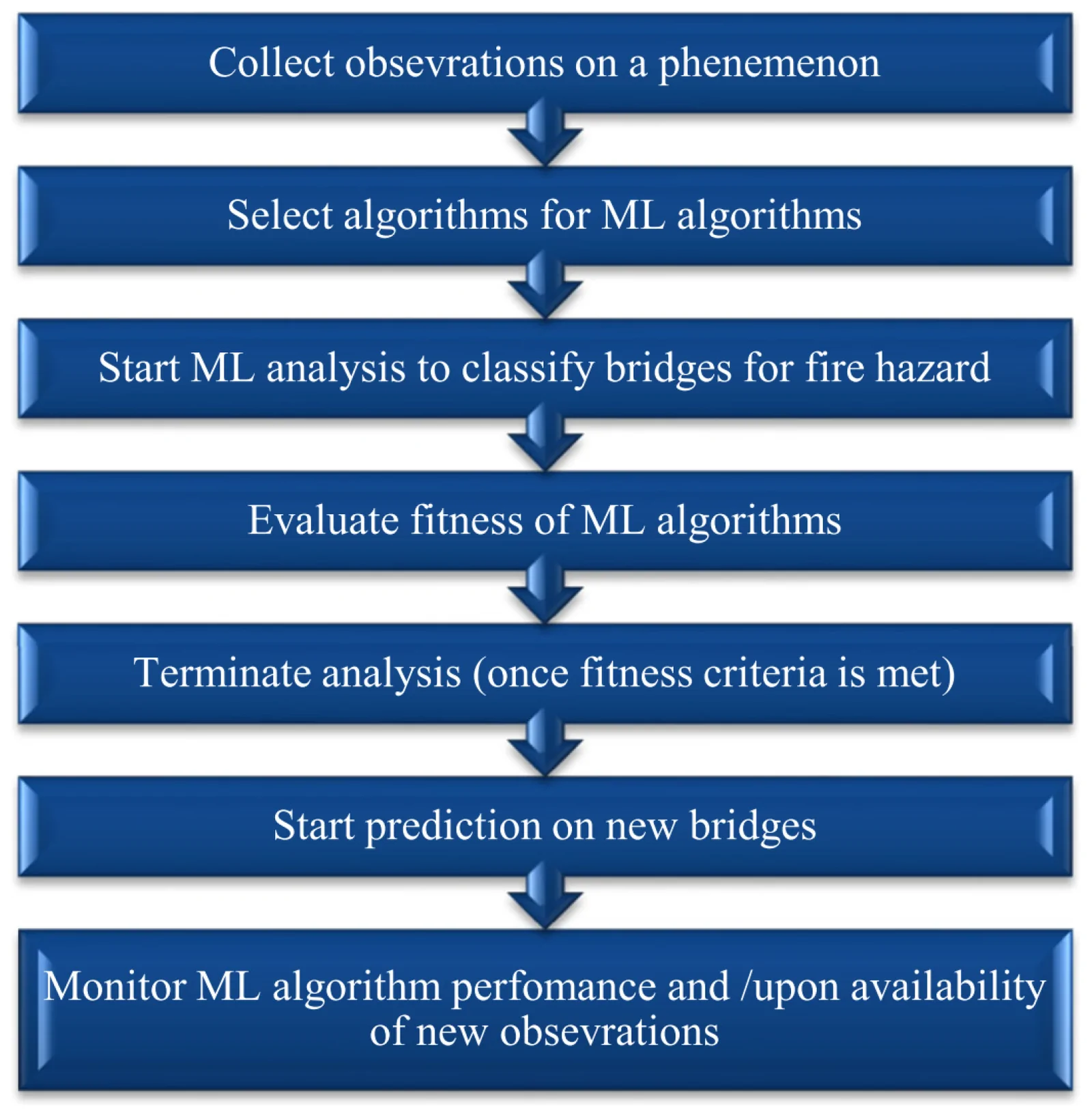
Bridge fire-risk assessment workflow based on competing machine-learning algorithms [83].

For infrastructure-level risk screening, Kodur and Naser analyzed fire incidents involving 80 steel bridges and 38 concrete bridges using random forest, support vector machines, and generalized additive models (GAMs). Input variables included bridge material, structural form, span length, age, number of traffic lanes, geographical importance, fuel type, and fire location. The overall classification accuracy was approximately 70%, and the different models consistently identified fuel type, span length, or bridge age as influential factors [83]. Figure 11 presents the machine-learning-based bridge fire-risk assessment workflow reported in the open-access study.

Another major limitation of machine learning for fire engineering is the scarcity of high-quality data. Naser et al. developed the StructuresNet and FireNet benchmark datasets and used them to compare decision trees, random forest, extreme gradient boosting, Light Gradient Boosting Machine (LightGBM), and deep-learning algorithms under consistent data and evaluation protocols. Their work emphasized that standardized datasets and unified evaluation procedures are prerequisites for meaningful comparisons among fire-performance models [84].

Table 8 summarizes representative machine-learning applications for elevated-temperature and fire-performance assessment of structural steels. Generative models may alleviate the shortage of full-scale fire-test data. However, the available representative study focused on reinforced-concrete columns and should therefore be regarded only as a methodological transfer case for data augmentation [85]. Synthetic data can increase sample density within the existing training distribution, but they cannot automatically generate buckling, connection failure, or progressive-collapse mechanisms that are absent from real experiments or reliable numerical simulations. Generative adversarial network (GAN)- and VAE-based augmentation must therefore be validated jointly against physics-based models and independent fire tests.

Current machine-learning studies on structural fire performance remain characterized by substantial disconnection across scales. Material-level models commonly neglect member boundary conditions and non-uniform temperature fields, member-level models rely heavily on numerical simulations, and structural-risk models often lack information on steel grade and fire-protection systems. A more rational direction is to connect temperature fields, material constitutive behavior, member failure, and structural-system response within a hierarchical framework and to propagate predictive uncertainty across each level, rather than developing isolated black-box models for individual stages.

### 5.3. Fatigue, Fracture, and Remaining-Life Assessment

Fatigue and fracture assessment of structural steels covers several distinct tasks, including fatigue-strength prediction, S-N curve construction, total fatigue-life prediction, low-cycle fatigue, welded-joint fatigue, corrosion fatigue, crack-growth-rate prediction, crack detection, and remaining-life estimation. These tasks differ in their input variables, target definitions, temporal scales, and evaluation criteria and therefore should not be compared solely by a common prediction error. In particular, fatigue-strength and S-N predictions generally focus on stress–life relationships, whereas crack-growth models predict da/dN, vision or nondestructive evaluation (NDE) models identify or quantify damage, and remaining-life models estimate the future failure horizon. This distinction is essential when assessing the engineering relevance of machine-learning (ML) models.

**Figure 12:**
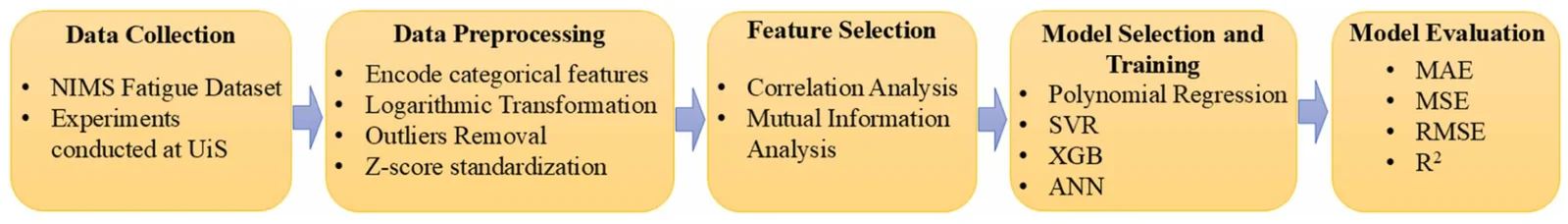
General workflow for machine-learning-based fatigue-life prediction of structural steels [88].

Early studies based on NIMS structural-steel fatigue data demonstrated that chemical composition and manufacturing parameters could be used to predict fatigue strength and identify influential variables [86,87]. Arvanitis et al. subsequently used NIMS data together with supplementary experiments to compare polynomial regression, support vector regression, XGBoost, and neural networks for structural-steel fatigue-life prediction. XGBoost achieved the lowest mean squared error, while polynomial regression remained competitive with neural networks at substantially lower computational cost [88]. This result indicates that, for structured fatigue datasets of moderate size, increasing model complexity does not necessarily lead to better engineering prediction. Figure 12 presents the general workflow for machine-learning-based fatigue-life prediction.

A critical issue in fatigue databases is the treatment of run-outs and right-censored observations. Specimens that have survived a prescribed number of cycles without failure do not provide an exact failure life and should not simply be discarded or treated as failed at the test limit. Their exclusion may bias the predicted high-cycle fatigue region, whereas assigning the test limit as an exact failure can distort the upper tail of the life distribution. Therefore, fatigue ML studies should explicitly report the number of run-outs, their censoring treatment, and, where appropriate, use censored regression or survival-analysis approaches. This consideration is particularly important when comparing models for total fatigue life and remaining-life prediction.

Welded joints require additional consideration because local geometry and manufacturing history strongly influence fatigue behavior. Relevant variables include joint class, weld geometry, weld-toe radius, defect type and size, residual stress, post-weld treatment, plate thickness, loading mode, stress ratio, mean stress, and variable-amplitude loading spectra. Feng et al. first transformed physical parameters from a welded-fatigue database and used XGBoost to determine feature weights before incorporating these weights into a deep convolutional neural network for S-N prediction [89]. Schubnell et al. further applied transfer learning from non-welded steel specimens to welded joints and compared the resulting predictions with conventional ML and fracture-mechanics-based approaches [90]. These studies indicate that transfer learning can reduce the data requirement of welded-joint prediction, but its effectiveness depends on whether the source and target domains share physically meaningful fatigue variables.

For engineering assessment, ML models should therefore be compared not only with alternative algorithms but also with established physical or design baselines. Depending on the task, appropriate baselines include S-N design curves, local stress/strain approaches, empirical fatigue relationships, Paris-law-type crack-growth models, and fracture-mechanics-based life assessment. Agreement with an ML model alone does not establish engineering validity; the additional value of ML should be demonstrated through improved prediction, broader applicability, reduced experimental requirements, or more efficient updating under new loading or environmental conditions.

Environmental effects further increase the variability of fatigue behavior. Feng et al. combined material strength, loading frequency, stress ratio, loading mode, temperature, and surface condition with Borderline-SMOTE, XGBoost, and an attention-based DCNN to predict corrosion-fatigue behavior [91]. The reported results show that incorporating environmental and mechanical variables can improve prediction under different test conditions. However, corrosion-fatigue models should distinguish between average degradation and localized damage because pits, corrosion defects, and crack-initiation sites may control structural failure even when the average corrosion rate remains relatively low. Validation should therefore preserve the chronological or site-specific structure of environmental data rather than relying solely on random record-level splitting.

Low-cycle fatigue provides another methodological transfer case. Jiang et al. incorporated temperature and strain rate into a physics-informed neural network for 316 stainless steels and introduced physical constraints into the loss function [92]. The predicted fatigue lives were reported within the twice-error band. Although this approach demonstrates the value of physical constraints for small datasets, the material-specific cyclic softening, creep-fatigue interaction, and temperature dependence of 316 stainless steel should not be assumed to be directly transferable to structural steels. The main methodological implication is that physical constraints can regularize ML models, but the constraints themselves must be reconstructed for the target steel and loading regime.

Fatigue-crack-growth prediction provides a direct connection between damage evolution and remaining-life assessment. Kamble et al. used carbon-steel compact-tension data to compare regression and nearest-neighbor approaches for predicting crack-growth behavior across both stable and rapid-growth regimes [93]. Such models extend beyond the conventional use of Paris-law-type relationships in the stable crack-growth region, but their engineering application still requires careful definition of the crack-size range, loading conditions, and fracture-mechanics variables. In particular, extrapolation beyond the experimentally covered crack-growth regime should not be interpreted as validated remaining-life prediction.

The development of image-based and NDE methods has further expanded ML from offline life prediction toward online damage detection. Long et al. used a global–local Faster R-CNN framework with images acquired during cyclic loading to identify small cracks and estimate crack length and growth rate [94]. Zhang et al. combined mechanoluminescent materials, computer vision, and ML for real-time fatigue-crack monitoring [95]. These studies demonstrate the potential of ML for surface-crack detection and quantitative monitoring. However, crack detection and remaining-life prediction are not equivalent tasks. Optical and other vision-based methods primarily provide information on visible surface damage and may not capture internal crack morphology. Future structural-steel applications should therefore combine multiple NDE modalities, such as ultrasonic testing, acoustic emission, magnetic methods, and X-ray computed tomography, where appropriate. Multimodal sensing may improve damage characterization, but the resulting measurements must still be linked to a physically meaningful damage state before they are used for prognostic life estimation [96].

A further methodological transfer case concerns hydrogen-assisted fatigue crack growth. For Cr-Mo steel exposed to gaseous hydrogen, gradient boosting combined with SHAP was used to evaluate the effects of stress-intensity-factor range, hydrogen pressure, strength, stress ratio, frequency, and chemical composition on crack-growth rate [97]. The study illustrates how ML can jointly represent material, loading, and environmental variables. However, the reported feature contributions should be interpreted only within the investigated Cr-Mo steel, hydrogen-pressure range, and loading conditions and should not be generalized as universal rankings for structural steels.

**Figure 13:**
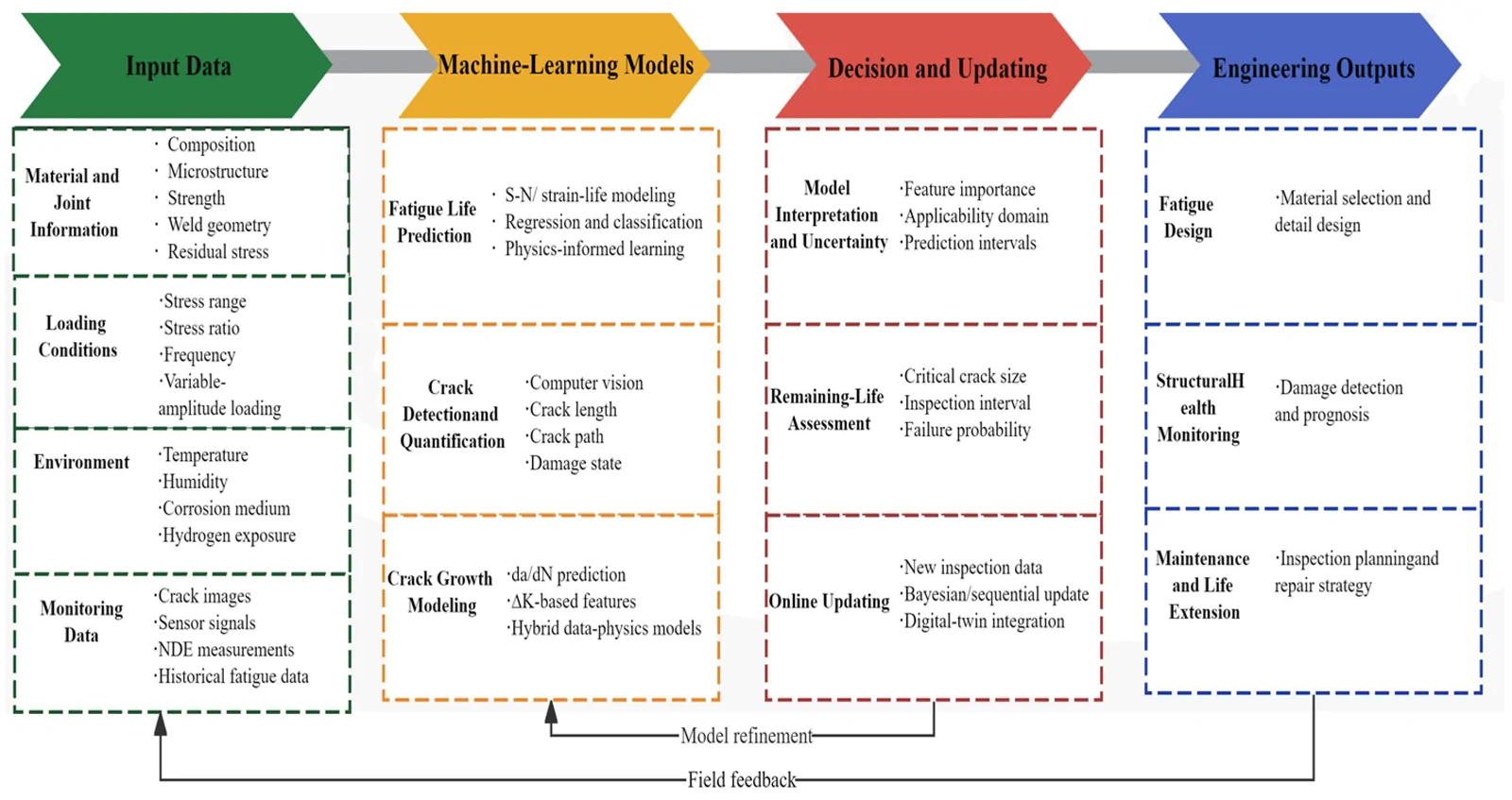
Integrated machine-learning framework for fatigue-life prediction, crack-growth monitoring, and remaining-life assessment of structural steels.

Another important issue is sample and sequence leakage. Fatigue datasets often contain repeated measurements from the same specimen, multiple images obtained during one test, or successive crack-growth observations from a single loading history. If these related observations are randomly divided between training and test sets, the model may partially recognize the specimen or test sequence rather than learn a transferable fatigue relationship. Therefore, specimen-level, test-level, or experiment-level grouping should be maintained during data partitioning, with all repeated observations from one specimen or test retained within the same partition. For crack-growth and image-based studies, this requirement is particularly important because adjacent measurements are strongly correlated. Figure 13 summarizes the continuous application chain of machine learning across fatigue-life prediction, crack-growth modeling, damage monitoring, and remaining-life assessment. Additional representative studies in this area are listed in Table 9.

Overall, ML applications in structural-steel fatigue have progressed from fatigue-strength and total-life prediction toward welded-joint assessment, environmental fatigue, crack-growth modeling, damage detection, and remaining-life estimation. However, these tasks require different target variables, baselines, validation strategies, and uncertainty treatments. The principal challenge is therefore not simply improving prediction accuracy, but establishing whether an ML model can provide a reliable engineering estimate under unseen specimens, loading histories, environments, and damage states. Future research should integrate fatigue databases, fracture-mechanics variables, multimodal NDE observations, and time-dependent monitoring data into dynamically updated remaining-life models.

## 6. Current Challenges and Future Directions

The studies reviewed in this study demonstrate that machine learning has extended from composition–property prediction to process optimization, microstructure characterization, inverse materials design, and durability and service-life assessment of structural steels. However, the principal barrier to further development is no longer whether high predictive accuracy can be achieved on an individual dataset, but whether the resulting models remain reliable when transferred across steel grades, production routes, laboratories, and service environments. For safety-critical structural steels, engineering deployment requires evidence of data independence, physical consistency, calibrated uncertainty, and performance beyond the original training domain.

### 6.1. Current Challenges

The first unresolved issue is the quality and independence of available evidence. Many structural-steel datasets contain large numbers of records but substantially fewer independent heats, specimens, production campaigns, exposure sites, or fatigue tests. As demonstrated in Section 3 and Section 4, internally randomized validation remains common, while independent cross-manufacturer, cross-laboratory, or field validation is much less frequent. Consequently, reported model accuracy often represents interpolation within an existing production or experimental domain rather than demonstrated engineering generalization. Data scarcity is particularly severe for fracture, localized corrosion, fire, corrosion fatigue, hydrogen-assisted cracking, and long-term monitoring, where the most safety-relevant observations are also the most difficult to obtain [97,98]. Inconsistent metadata, interoperability, and reporting practices further restrict reliable reuse and cross-source integration of materials data [99,100,101].

A second challenge is the limited connection between statistical prediction and physical state evolution. Composition and process variables alone may be sufficient for interpolation within a narrow steel family, but they do not uniquely describe microstructure, defects, residual stress, environmental damage, or crack evolution. Conversely, image-based and sensor-based models can capture local information but remain sensitive to specimen preparation, acquisition conditions, labeling, and spatial or temporal correlation. The major unresolved problem is therefore not simply multimodal data fusion, but the construction of scale-bridging state variables that connect composition, processing, microstructure, defects, local damage, and structural response without losing physical interpretability [102].

The third challenge concerns uncertainty and engineering conservatism. Most reported models still provide deterministic predictions and average performance metrics, whereas structural-steel applications require knowledge of whether an individual prediction is sufficiently reliable for a safety-related decision. Aleatoric variability in material properties and epistemic uncertainty caused by sparse training data must be distinguished. Moreover, prediction errors are direction-sensitive: overestimating fatigue life or member resistance can be more critical than an error of the same magnitude in the conservative direction. Applicability-domain assessment, calibrated prediction intervals, out-of-distribution detection, and explicit reporting of non-conservative errors therefore remain essential gaps between academic model accuracy and engineering reliability [103].

Finally, verification, governance, and deployment procedures remain immature. Many studies terminate after internal model testing, whereas few demonstrate prospective alloy production, independent manufacturing-line validation, full-scale structural testing, or long-term field deployment. Model updates caused by new steel grades, process drift, sensor replacement, or changing environmental conditions are rarely governed by predefined procedures. Version control, traceable audit records, responsibility for model updates, conservative fallback rules, cybersecurity of industrial data, and compatibility with existing material specifications and structural design standards must therefore be treated as part of model reliability rather than as downstream software issues.

### 6.2. Future Research Directions

In the near term, priority should be given to standardized data and reporting practices and to stronger validation protocols. Structural-steel datasets should preserve heat number, product form, thickness, sampling position and orientation, manufacturing and welding history, specimen geometry, testing standard, environmental conditions, censoring information, measurement uncertainty, and data-processing lineage. Reporting should distinguish raw records from statistically independent material units. Validation should progress from random splitting toward heat-, specimen-, source-, site-, and production-based grouping, followed where possible by independent manufacturer or laboratory data. Success should be assessed not only through lower RMSE or higher R^2^, but through predefined criteria such as successful external validation, calibrated prediction-interval coverage, out-of-distribution detection performance, and the frequency of non-conservative errors.

In the medium term, research should focus on physics-informed, multi-fidelity, transfer-learning, and uncertainty-aware frameworks. CALPHAD, phase-transformation kinetics, strengthening models, finite-element analysis, corrosion transport, and fracture mechanics can provide intermediate state variables, physical constraints, or lower-fidelity information, while machine learning can represent relationships that remain difficult to formulate explicitly [104,105,106]. Transfer learning should be accepted only when the source and target domains share identifiable physical information, and simulation-derived data should be calibrated against experimental observations rather than pooled indiscriminately with them. Active learning can further reduce experimental demand by selecting experiments with high expected information gain [107,108]. For materials design, a meaningful success criterion is not merely rediscovery of known alloys but prospective validation of genuinely new candidates, together with evidence of manufacturing robustness and reduced experimental campaigns relative to conventional search strategies.

Closed-loop experimentation represents a further medium-term objective. Systems such as CAMEO demonstrate how active learning, physical knowledge, and rapid characterization can iteratively update material-selection decisions [109]. For structural steels, closed-loop development should initially focus on laboratory alloy design, heat-treatment windows, welding parameters, and high-cost corrosion or fatigue experiments. Model-generated candidates should remain subject to expert constraints on manufacturability, weldability, cost, standards compliance, and safety. A closed loop should therefore be evaluated by the number of experiments required to reach a validated design, its ability to identify candidates outside the initial dataset, and the reproducibility of the resulting material rather than solely by optimization speed.

**Figure 14:**
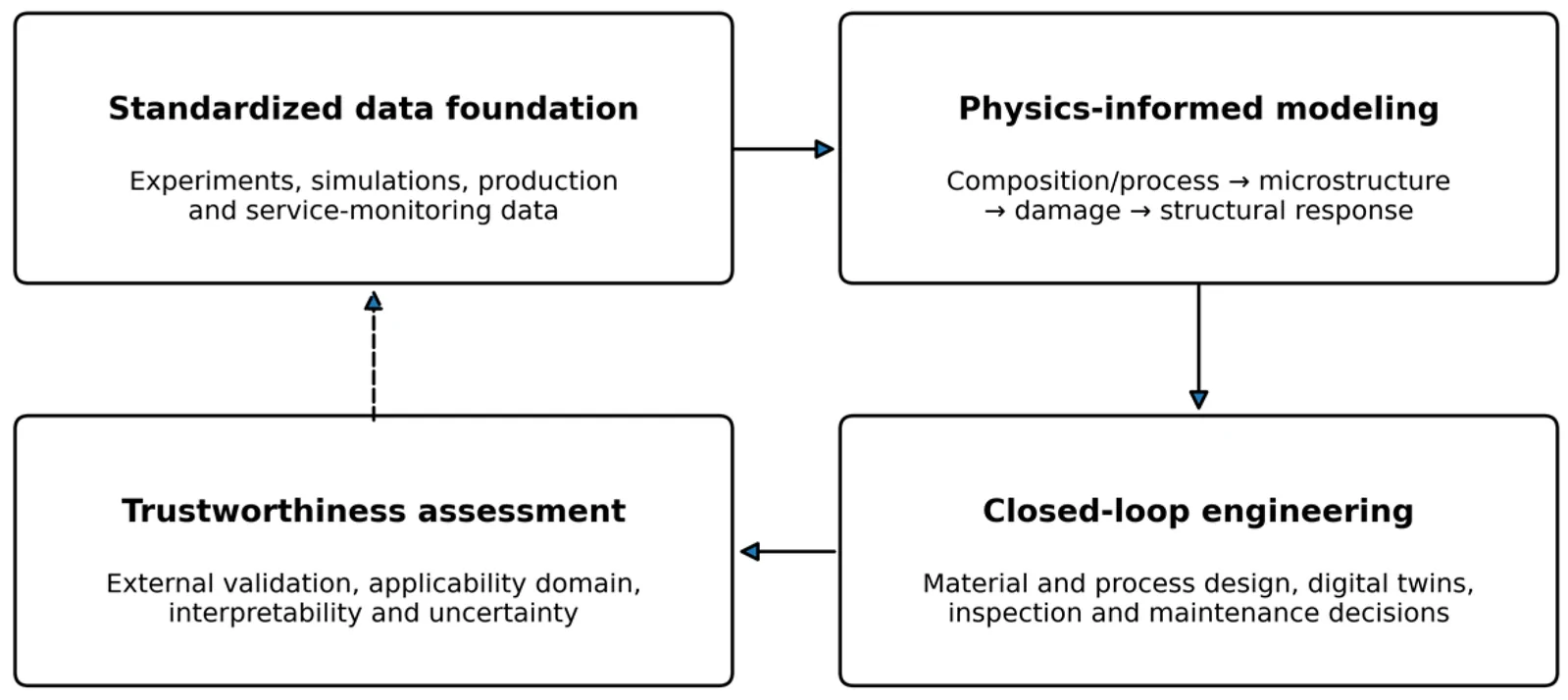
Roadmap for trustworthy machine learning in structural-steel design, manufacturing, and in-service management.

In the long term, digital twins could connect steel manufacturing history with evolving in-service damage and maintenance decisions. In this context, a structural-steel digital twin should be defined operationally rather than as a generic digital representation. Relevant state variables may include microstructure, local mechanical properties, residual stress, corrosion depth, pit geometry, crack size, and accumulated fatigue damage. Sensor inputs may include temperature, strain, corrosion measurements, ultrasonic or acoustic-emission signals, and crack-monitoring data. The twin should specify when state estimates are updated, how model parameters are recalibrated after new inspections, how uncertainty is propagated from sensing to remaining-life prediction, and which engineering decision is supported—for example, continued service, inspection scheduling, repair, or replacement. A useful digital twin must therefore demonstrate that dynamic updating improves decision reliability relative to a static design-stage life estimate [110]. Figure 14 presents a roadmap for trustworthy machine-learning deployment across structural-steel design, manufacturing, and in-service management.

Long-term deployment will also require explicit model governance and integration with engineering standards. Each deployed model should have a controlled version, traceable training and validation data, an audit trail of changes, assigned responsibility for approval and updating, predefined out-of-domain and sensor-failure responses, and a conservative fallback to an accepted physical model or design provision when confidence is insufficient. Cybersecurity and access control are particularly relevant when production records and field-monitoring data are incorporated into continuously updated models.

The National Institute of Standards and Technology (NIST) Artificial Intelligence Risk Management Framework should therefore be regarded as a general governance reference rather than a structural-steel design standard [111]. Its principles can be translated into materials-specific verification activities: governance corresponds to model ownership, version control, and auditability; mapping corresponds to defining the steel grade, process, service condition, and consequence of failure; measurement corresponds to independent validation, calibration, applicability-domain assessment, and non-conservative error analysis; and management corresponds to model updating, out-of-distribution handling, conservative fallback, and retirement. Regulatory adoption will require these procedures to be connected to existing material qualification and structural-design practices rather than replacing established engineering standards with a generic ML model. The specific research gaps that need to be addressed for each of these areas are listed in Table 10.

Overall, the development pathway is expected to progress from standardized and independently validated datasets in the near term, through physics-informed and uncertainty-aware models with prospective experimental validation in the medium term, toward closed-loop material development, operational digital twins, and code-compatible model governance in the long term. The central measure of progress should therefore shift from dataset-specific prediction accuracy to demonstrated reductions in uncertainty, experimental burden, non-conservative prediction, and life-cycle decision risk.

## 7. Conclusions

This review shows that machine learning has become a useful complementary tool for structural-steel research across composition and process modeling, microstructure characterization, mechanical-property prediction, inverse design, and durability assessment. The available evidence indicates that tree-based methods are frequently effective for small- to medium-sized tabular datasets, whereas image- and sequence-based tasks increasingly employ deep-learning approaches. However, model performance is strongly dependent on dataset composition, feature representation, and validation strategy; no algorithm can therefore be regarded as universally superior across structural-steel applications.

The maturity of the available evidence differs substantially among application areas. Composition, processing, and mechanical-property prediction are comparatively well developed, and a limited number of alloy- and process-design studies have progressed to experimental validation of newly selected candidates. Some industrial-property models have also been evaluated using previously unseen production samples. In contrast, corrosion, fire, fatigue, crack-growth, and remaining-life models are still dominated by internal validation, literature-derived datasets, laboratory-scale experiments, or simulation-based evidence. Prospective cross-manufacturer validation, independent production-line testing, full-scale structural verification, and long-term field deployment remain uncommon. Consequently, many service-life applications should presently be regarded as proof-of-concept or decision-support tools rather than validated replacements for established engineering models or design provisions.

Across the reviewed studies, the most persistent limitation is not predictive accuracy itself but the strength of the supporting evidence. Large record counts often do not correspond to equally large numbers of independent heats, specimens, joints, or exposure campaigns, and random data partitioning can obscure this distinction. For safety-critical use, future progress should therefore be judged by independent validation, calibrated uncertainty, applicability-domain control, physically meaningful baselines, and the rate of non-conservative predictions rather than by R^2^, RMSE, or classification accuracy alone. Physics-informed descriptors, multi-fidelity learning, and multimodal monitoring are promising only when their added complexity produces demonstrable improvements in external generalization or engineering decision quality.

This review also has limitations. The synthesis is affected by publication and literature-selection bias, heterogeneity in prediction targets and performance metrics, and incomplete reporting of independent sample numbers, testing conditions, uncertainty, and external validation in many primary studies. In addition, selected adjacent steel systems and methodological transfer cases were included to illustrate approaches not yet sufficiently demonstrated in conventional civil-infrastructure structural steels; these examples should not be interpreted as direct evidence of structural-steel readiness. Overall, the field has progressed beyond proof-of-principle property fitting, but widespread engineering deployment will require a shift from dataset-specific accuracy toward independently validated, uncertainty-aware, and physically defensible models.

## Figures and Tables

**Table 1:** Comparison of the present review with representative previous reviews on machine learning in materials and structural engineering.

Review Category	Main Material or Structural Scope	Major Data Modalities	Materials Design and Property Prediction	Service Degradation and Life Assessment	Trustworthiness and Engineering Validation
Alloy and metallic-material ML reviews [4,5,6]	Broad alloy and metallic systems	Composition, computation, tabular data	Extensive	Limited	Partially addressed
Microstructure and inclusion reviews [2,13]	Steels and metallic materials	Microscopy and image data	Specialized	Limited	Limited
Constitutive and fatigue reviews [14,15,16]	Metallic materials and steels	Mechanical and fatigue data	Property-specific	Primarily fatigue-related	Partially addressed
Building-material ML reviews [17]	Concrete and functional construction materials	Mixed	Broad	Partial	Limited
Steel-structure AI reviews [18]	Members and structural systems	Structural, numerical, and monitoring data	Limited at the material scale	Structural-performance oriented	Partially addressed
Steel manufacturing and performance reviews [19,20,21,22]	Steel production and materials	Industrial and process data	Extensive	Limited	Partially addressed
Present review	Civil-infrastructure structural steels, with explicitly classified adjacent and transfer evidence	Tabular, imaging, three-dimensional, simulation, sensor, and service data	Integrated from composition and process design to microstructure and property prediction	Corrosion, fire, fatigue, fracture, and remaining life	Data independence, physical consistency, interpretability, uncertainty, applicability domain, and external validation

**Table 2:** Major data types used in machine learning for structural steels.

Data Type	Representative Data Scale and Independent Unit	Essential Metadata	Accessibility	Recommended Partitioning Unit	Principal Limitation	Ref.
Composition and processing data	Large numbers of records may originate from substantially fewer independent heats or production campaigns	Steel grade, heat number, product form, thickness, complete processing route, equipment state	Mainly proprietary; some literature and public datasets	Heat, steel grade, production campaign, or chronological period	Strong collinearity, process drift, and incomplete processing histories	[19,20,21,22]
Mechanical and durability data	Usually limited by the number of independently tested specimens, conditions, or exposure series	Specimen geometry, sampling orientation, test standard, temperature, strain/loading rate, environment, run-out/censoring status	Literature, laboratory databases, selected public repositories	Specimen, heat, laboratory, or exposure condition	High testing cost, censoring, and scarcity of failure or boundary-condition data	[19,20]
Microstructural images	Image or patch counts may substantially exceed the number of original specimens; e.g., 1705 SEM images and 8909 annotated regions in Aachen–Heerlen	Specimen ID, imaging modality, magnification, pixel size, preparation, field of view, labeling protocol	Several open repositories available	Original specimen or independently acquired field of view	Label uncertainty, imaging-scale dependence, and patch-level leakage	[2,23,24]
Defect and three-dimensional data	Multiple defects may originate from a small number of independently tested volumes or specimens	Specimen ID, voxel size, reconstruction settings, detection threshold, loading history, defect definition	Limited but increasing open availability	Original specimen or scanned volume	High acquisition cost and sensitivity to reconstruction and thresholding	[13,25]
Industrial time-series and sensor data	Very large numbers of time points may originate from relatively few production campaigns	Sensor type, sampling frequency, calibration state, equipment condition, heat/campaign ID, maintenance events	Predominantly proprietary	Production campaign, heat, or chronological block	Autocorrelation, sensor drift, operating-condition change, and leakage	[20,21,22]
Numerical simulation data	Potentially large parameter-space coverage; independent unit is a simulated physical state rather than an experimental specimen	Model fidelity, governing equations, material parameters, boundary conditions, mesh/resolution, calibration and uncertainty	Often reproducible when models and inputs are available	Parameter-space region or physical scenario	Model-form discrepancy, parameter uncertainty, and simulation-to-experiment bias	[3,20,28]

**Table 3:** Trustworthy evaluation framework for machine-learning models of structural steels.

Evaluation Dimension	Core Question	Recommended Evaluation Approach	Ref.
Predictive accuracy	Can the model accurately describe known samples?	R^2^, MAE, RMSE, F1 score, AUC, and region-specific errors	[19,35]
Physical consistency	Are the predicted trends consistent with metallurgical and mechanical principles?	Verification against boundary conditions, variable trends, and physics-based models	[3,31,39]
Generalization	Can the model be transferred to new heats, steel grades, and service conditions?	Leave-one-heat-out, leave-one-grade-out, cross-manufacturer, and external validation	[36,37]
Uncertainty	Is an individual prediction sufficiently reliable?	Prediction intervals, coverage probability, and calibration error	[38]
Interpretability	Does the model identify physically reasonable controlling factors?	SHAP analysis, sensitivity analysis, and partial-dependence plots	[39]
Reproducibility	Can the data, model, and results be independently reproduced?	Public availability of data, code, metadata, and partitioning protocols	[29,30,35]

**Table 4:** Representative applications of machine learning to composition, processing, and microstructure design of structural steels.

Material or Application Scope	Data and Variables	Models and Validation	Key Findings, Validation Evidence, and Applicability Limitations	Ref.
Ultrahigh-strength stainless steel as a methodological transfer case	Small literature-derived and experimental dataset; composition, aging conditions, and precipitation-related physical variables used to predict hardness and feasibility	Physics-guided SVM, classifier, and GA; cross-validation and independent alloy fabrication	Physics-based features excluded infeasible candidates; the material is not a conventional civil-engineering structural steel	[40]
Phase transformations in high- and low-alloy steels	Literature and experimental TTT and CCT data; composition and cooling conditions used to predict transformation products, temperatures, and hardness	Ensemble regression, multilayer perceptron (MLP), RF, and symbolic regression; test-set evaluation and comparison with conventional models	Accelerated prediction of transformation diagrams; limited consistency across databases	[41,42]
Hardenability of non-boron and boron steels	Jominy-test data; composition and distance from the quenched end used to predict hardness profiles and optimize composition	RF, k-nearest neighbors (kNN), and combined models; cross-validation and validation of candidate steels	Suitable for curve-valued outputs; performance depends on the coverage of compositionally similar steels	[43,44]
Complex steel microstructures	SEM and EBSD images with limited numbers of original specimens; image-based phase classification, segmentation, and quantification	Fully convolutional neural network (FCNN), U-Net, and other deep segmentation models; specimen-level partitioning and cross-grade testing	EBSD-derived labels improved physical fidelity; performance remained sensitive to specimen preparation and magnification	[46,47,48,49]

**Table 5:** Representative machine-learning studies for mechanical-property prediction of structural steels.

Property	Data Basis	ML Approach and Validation	Main Limitation	Ref.
Tensile properties	Industrial production data	Tree-based/ensemble models; group-level validation	Independent heats should be clarified	[51,52]
Tensile properties	63,137 records	RF/regression; internal validation	Records may not represent independent heats	[50]
Charpy impact energy	Literature and industrial data	Regression/ensemble; test-set validation	Temperature and specimen conditions vary	[53,54]
Elevated-temperature properties	High-temperature experiments	Regression/ensemble; experimental validation	Heating and loading histories vary	[55,56]

**Table 6:** Principal machine-learning tasks in structural-steel durability and service-life assessment.

Task	Main Target	Typical Scale
Corrosion-rate prediction	Corrosion rate/current	Material
Localized-damage prediction	Pit depth/morphology	Local surface
Elevated-temperature degradation	Retained properties	Material
Fire-resistance prediction	Member resistance	Component
Fatigue-strength prediction	Fatigue strength	Material/joint
Total-life prediction	Fatigue life	Material/component
Crack-growth monitoring	Crack length/growth rate	Local/component
Remaining-life estimation	Residual service life	Component/structure

**Table 7:** Representative machine-learning studies for corrosion and environmental degradation of structural steels.

Exposure Class	Study	Target (Unit)	Exposure/Time	Model and Validation	Localized Damage	Ref.
Atmospheric	Carbon steel sensor	Instantaneous galvanic current (μA)	34 d	RF; time-series testing	No	[69]
Marine atmosphere	Low-alloy steels	Corrosion rate (μm·a^−1^)	1–10 yr	RF; train/test split	No	[70]
Dynamic atmosphere	Carbon steel	Corrosion rate (μm·a^−1^)	Dynamic vehicle exposure	GA-SVR; hold-out testing	No	[71]
Seawater	3C steel	Corrosion current density (μA·cm^−2^)	Multiple seawater conditions	ANN; unseen test data	No	[71]
Soil/underground	Steel	Corrosion current density (A·m^−2^)	0–415 d	RF; random 80/20 split	No	[73]
Steel–concrete	Steel in mortar	Corrosion current density (μA·cm^−2^)	Laboratory exposure	SVR; cross-validation	No	[74]
High-temperature corrosion	Structural/coating materials	Corrosion rate (study-specific units)	Heterogeneous literature data	RF; internal validation	Not resolved	[75]
Pipeline/service life	Pipeline systems	Time to failure (source-defined time unit)	Historical failure records	Extra Trees; CV + test set	Not resolved	[76]
Marine pipe piles	Steel pipe piles	Corrosion depth (mm); service life (yr)	Accelerated test + long-term extrapolation	PINN; experimental validation	No; uniform corrosion assumption	[77]

Note: “Localized damage” indicates whether the machine-learning target explicitly represents spatially concentrated corrosion such as maximum pit depth or pit morphology. “No” indicates that the model primarily predicts an average or electrochemical corrosion quantity. “Not resolved” indicates that localized damage was not explicitly represented in the reviewed model.

**Table 8:** Representative applications of machine learning to elevated-temperature and fire-performance assessment of structural steels.

Scale or Application Scope	Data and Variables	Models and Validation	Key Findings and Limitations	Ref.
Material	Multisource elevated-temperature material data; temperature and material parameters used to predict thermal-property and mechanical-property reduction	ANN combined with GA; comparison with code provisions and experiments	Heterogeneous data could be integrated, but definitional inconsistencies propagated into member-level calculations	[78]
Material	More than 450 expanded records; temperature and plate thickness used to predict elevated-temperature stress–strain behavior	Gradient boosting, RF, XGBoost, and SVR; experimental validation	Gradient boosting achieved an adjusted (R^2^ > 0.98); extrapolation beyond the calibrated temperature range requires caution	[79]
Member	Experimental and analytical steel-column data; temperature, geometry, and loading variables used to predict compressive resistance	Hybrid neural network; comparison with analytical equations and experiments	Capable of handling multiple interacting variables; coverage of the early dataset was limited	[80]
Member	21,879 GMNIA samples; cross-sectional geometry, temperature, and slenderness used to predict fire resistance	Neural network, RF, and SVM with SHAP analysis; independent test set	Suitable as a rapid surrogate model; bias inherited from numerical simulation remained unresolved	[81]
Structural system	Monte Carlo-generated fire scenarios; temperature and loading variables used to predict member failure and structural collapse	Decision tree, KNN, and ANN; validation using numerical case studies	Linked member failure with system-level response; full-scale experimental evidence remained limited	[82]
Infrastructure risk	Fire records for 118 bridges; structural and fire-related features used for risk classification	RF, SVM, and GAM; cross-validation	Classification accuracy was approximately 70%; the dataset was heterogeneous and limited in size	[83]
Benchmarking and data augmentation	FireNet and synthetic test data; multiple inputs used to predict fire performance	Benchmark models, GAN, and VAE; unified benchmarking and internal validation	Improved model comparability; synthetic data could not generate previously unobserved failure mechanisms	[84,85]

**Table 9:** Representative applications of machine learning to fatigue, fracture, and remaining-life assessment of structural steels.

Task/Scope	Data Characteristics	ML Approach	Validation and Data Treatment	Main Limitation	Ref.
Fatigue-strength prediction	NIMS steel fatigue data; composition and processing variables	Regression and ensemble models	Cross-validation; independent specimen number and run-out treatment not reported	Applicability restricted by database coverage	[86,87]
Total fatigue-life prediction	NIMS data and supplementary fatigue tests	Polynomial regression, SVR, XGBoost, ANN	Train/test evaluation; specimen independence and censored-data treatment not fully reported	External validation remains limited	[88]
Welded-joint S–N prediction	Weld geometry, loading conditions, and physics-based descriptors	XGBoost and DCNN	Cross-validation; uncertainty not explicitly quantified	Transferability across joint classes and loading spectra remains limited	[89]
Welded-joint fatigue transfer	22 target-domain welded-joint datasets	Transfer learning	Target-domain validation and comparison with conventional models	Performance depends on similarity between source and target domains	[90]
Corrosion-fatigue life	Multisource data covering stress ratio, frequency, temperature, and environment	XGBoost and attention-based DCNN	Cross-condition evaluation; prediction intervals not reported	Validation under realistic variable-amplitude loading remains insufficient	[91]
Low-cycle fatigue, methodological transfer case	316 stainless steel under different temperatures and strain rates	Physics-informed neural network	Factor-of-two error-band evaluation	Physical constraints require reformulation for structural steels	[92]
Fatigue-crack-growth prediction	Carbon-steel compact-tension data; fracture-mechanics variables	Regression and KNN	Experimental test-data evaluation; uncertainty not reported	Extrapolation beyond the tested crack-growth regime is uncertain	[93]
Crack detection and monitoring	Cyclic-loading image sequences; surface crack length and growth rate	Faster R-CNN and vision-based ML	Experimental sequence evaluation; specimen-level independence should be preserved	Primarily limited to visible surface cracks	[94,95]
Hydrogen-assisted crack growth, methodological transfer case	Cr–Mo steel; hydrogen pressure, loading and material variables	Gradient boosting and SHAP	Train/test evaluation within the investigated material and loading domain	Results should not be generalized beyond the specified Cr–Mo steel and hydrogen conditions	[96]

Note: NIMS, National Institute for Materials Science; SVR, support vector regression; ANN, artificial neural network; DCNN, deep convolutional neural network; KNN, k-nearest neighbors; R-CNN, region-based convolutional neural network; SHAP, SHapley Additive exPlanations. Where the original study did not explicitly report the number of statistically independent specimens, treatment of fatigue run-outs, or prediction uncertainty, this limitation is stated rather than inferred.

**Table 10:** Priority research gaps and readiness of machine learning for structural steels.

Gap	Priority Action	Horizon	Current Readiness	Evidence Basis
Heterogeneous data	Standardize metadata and provenance	Near term	Developing	Inconsistent datasets
Limited generalization	Grouped and external validation	Near term	Developing	Mainly internal validation
Uncertainty/OOD	Calibrated intervals and OOD detection	Near–medium	Early	Limited uncertainty reporting
Physical consistency	Physics-informed and multi-fidelity models	Medium term	Developing	Narrow physical integration
Experimental cost	Active learning and transfer learning	Medium term	Early–developing	Small high-cost datasets
Inverse design	Prospective candidate validation	Medium term	Early	Mainly limited experimental validation
Dynamic life assessment	Operational digital twins	Long term	Early	Weak cross-scale updating
Engineering deployment	Governance and code integration	Long term	Early	Limited deployment evidence

## Data Availability

No new data were created or analyzed in this study. Data sharing is not applicable to this article.
